# SPAT inhibits LUAD metastasis by targeting SF1-mediated splicing

**DOI:** 10.1038/s41419-025-07924-2

**Published:** 2025-08-08

**Authors:** Yun Ma, Xiaoxu Zhou, Mengqian Yu, Xiang Cheng, Juze Yang, Jiayi Ren, Chengcai Zheng, Jia Li, Xinyi Qian, Jiani Yi, Honghe Zhang, Yan Lu, Pengyuan Liu

**Affiliations:** 1 Department of Respiratory Medicine, Sir Run Run Shaw Hospital and Institute of Translational Medicine, Zhejiang University School of Medicine, Hangzhou, Zhejiang China; 2 Zhejiang Provincial Key Laboratory of Precision Diagnosis and Therapy for Major Gynecological Diseases, Women’s Hospital and Institute of Translational Medicine, Zhejiang University School of Medicine, Hangzhou, Zhejiang China; 3 Department of Pathology, Research Unit of Intelligence Classification of Tumor Pathology and Precision Therapy, Chinese Academy of Medical Sciences, Zhejiang University School of Medicine, Hangzhou, Zhejiang China; 4 Laboratory of Frontier Medical Research on Cancer Metabolism, Zhejiang University School of Medicine, Hangzhou, Zhejiang China

**Keywords:** Non-small-cell lung cancer, Long non-coding RNAs, Prognostic markers

## Abstract

Lung adenocarcinoma (LUAD) progression involves alterations in oncogenes and tumor suppressor genes, collectively shaping tumorigenic landscape. However, the precise interactions within this landscape remain inadequately understood. Here, we present a functional characterization of a novel long non-coding RNA (lncRNA), SPAT (splice associated transcript). SPAT is downregulated in LUAD and its expression positively correlates with favorable prognosis. In vitro and in vivo experiments demonstrated that SPAT inhibits the migration of LUAD cells. This inhibitory effect is mediated by SPAT’s interaction with splicing factor 1 (SF1), which disrupts SF1-mediated splicing of KITLG/SCF exon 6, thereby suppressing ERK phosphorylation. Our findings suggest that SPAT acts as a tumor suppressor in LUAD by regulating alternative splicing and highlight its potential as a therapeutic target for managing LUAD metastasis.

**Figure:**
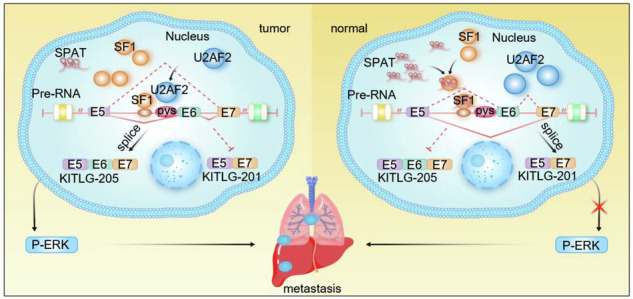
SPAT suppresses LUAD cell migration by binding to splicing factor 1 (SF1) and disrupting SF1-mediated inclusion of exon 6 in the KITLG/SCF transcript. This shifts KITLG splicing toward increased production of KITLG-201 isoform and reduced KITLG-205, ultimately lowering ERK phosphorylation and limiting metastatic potential.

SPAT suppresses LUAD cell migration by binding to splicing factor 1 (SF1) and disrupting SF1-mediated inclusion of exon 6 in the KITLG/SCF transcript. This shifts KITLG splicing toward increased production of KITLG-201 isoform and reduced KITLG-205, ultimately lowering ERK phosphorylation and limiting metastatic potential.

## Introduction

Lung cancer remains the leading cause of cancer-related incidence and mortality worldwide, with the World Health Organization (WHO) reporting ~2,480,308 new cases and 1,817,131 deaths in 2022 alone [1, 2]. Among its histological subtypes, lung adenocarcinoma (LUAD) stands out as the predominant type, constituting around 40% of all lung cancer cases [3]. Despite advancements in targeted therapy and immunotherapy, alongside conventional treatments, achieving a satisfactory 5-year survival rate for LUAD remains a formidable challenge [4]. This underscores the critical need for further exploration of the molecular mechanisms driving LUAD pathogenesis and the identification of novel diagnostic and therapeutic biomarkers.

Long noncoding RNAs (lncRNAs), a subclass of noncoding RNAs (ncRNAs) longer than 200 nucleotides and lacking protein-coding capacity, have emerged as key players in various cancers, including LUAD [5, 6]. Numerous studies have demonstrated that dysregulated lncRNA expression is a prevalent phenomenon implicated in LUAD pathogenesis [7, 8]. For instance, lncRNA LCAT1 promotes LUAD growth and metastasis by stabilizing the IGF2BP2-CDC6 axis [9], while m6A-regulated LCAT3 enhances lung cancer progression through its interaction with FUBP1 to activate c-MYC [10]. Additionally, LINC00261, an epigenetically regulated tumor suppressor, plays a crucial role in activating the DNA damage response in LUAD [11]. Despite these notable findings, our understanding of the biological roles and mechanisms of many lncRNAs in LUAD remains rudimentary.

Pre-mRNA splicing is a crucial process in eukaryotic gene expression, with alternative splicing (AS) allowing a single gene to produce multiple transcripts, thereby increasing protein diversity and influencing phenotypic traits [12]. The spliceosome, a complex assembly of five small nuclear ribonucleoprotein particles (U1, U2, U4, U5, and U6 snRNPs) and over 100 non-snRNP proteins, orchestrates the precise removal of introns from pre-mRNA through intricate RNA–RNA, RNA–protein, and protein–protein interactions [13]. Different combinations of exons in pre-mRNA give rise to distinct mature mRNAs via five primary AS patterns: exon skipping (ES), retained intron (RI), mutually exclusive exons (ME), alternative 5’ splice site (A5SS), and alternative 3’SS (A3SS) [14]. Growing evidence suggests that disruptions in splicing can contribute to cancer by altering the expression of splicing machinery components [15, 16]. For instance, Cyclin D1 (CCND1) undergoes alternative splicing to generate cyclin D1b isoform lacking Thr-286. Unlike the canonical cyclin D1a isoform, cyclin D1b is highly expressed in many tumors and exhibits tumorigenic properties [17]. Additionally, SRSF6 promotes proliferation and metastasis in colorectal cancer by directly binding to the ZO-1 motif within exon 23 [18].

In this study, we identified a novel LUAD-associated lncRNA, SPAT, which is downregulated in LUAD samples. Its expression is positively associated with favorable prognosis in LUAD patients. Loss of SPAT significantly facilitated LUAD cell metastasis, whereas upregulation of SPAT expression suppressed LUAD cell migration. Mechanistic analysis revealed that SPAT physically interacts with SF1, thereby blocking SF1 recognition of the KITLG/SCF exon 6 branch site. This interaction modulates a major AS event, leading to the reduction of the protein-coding transcript KITLG-205, which facilitates LUAD cell metastasis by regulating ERK phosphorylation. These findings provide novel insights into the intricate mechanisms driving LUAD progression and suggest SPAT as a potential therapeutic target and prognostic biomarker for LUAD.

## Result

### Low expression of SPAT is associated with poor prognosis in LUAD

**Fig. 1:**
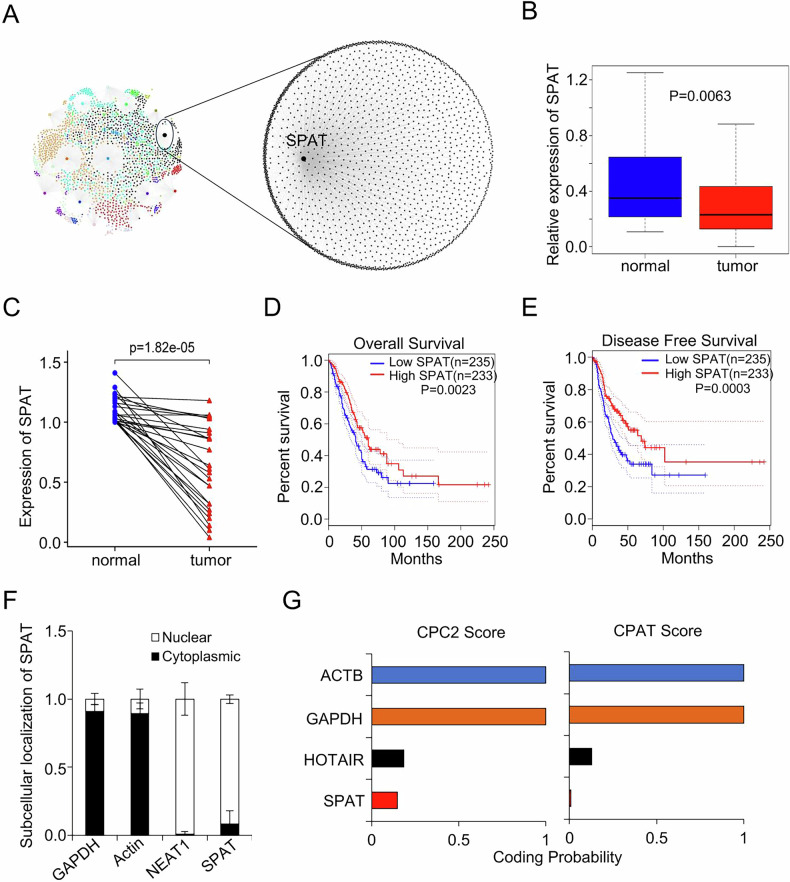
Low expression of SPAT is associated with a poor prognosis in LUAD. **A** LUAD co-expression network. Co-expression networks for two conditions (tumor and normal) were constructed based on the reference mRNA-lncRNA interaction network, respectively. Each node of the co-expression network represents a mRNA gene or lncRNA, and the thickness of each edge represents the magnitude of Pearson correlation coefficient of two nodes. **B** Relative expression of SPAT in LUAD tissues and adjacent normal tissues in TCGA cohort. **C** qPCR analysis of SPAT expression in an independent cohort of 25 pairs of LUAD tissues and matched adjacent normal tissues. Kaplan–Meier analysis of overall survival (**D**) and disease-free survival (**E**) of LUAD patients from the TCGA datasets. **F** qPCR analysis of cytoplasmic or nuclear SPAT RNA levels in A549 cells. GAPDH served as the cytoplasmic control, and NEAT1 as the nuclear control. **G** The coding potential of SPAT was evaluated using CPC2 (Coding Potential Calculator 2) and CPAT (Coding Potential Assessment Tool), HOTAIR was the negative control, whereas ACTB and GAPDH were the positive controls.

To illuminate the evolutionary trajectory of LUAD, we constructed a lncRNA co-expression network using WMDS.net [19], focusing on 15 lncRNAs with significant differential expression between LUAD and adjacent normal tissues. Through in silico analysis and functional screening, SPAT emerged as a critical regulator of the transcriptomic network, warranting further investigation (Fig. 1A and Supplementary Fig. S1A). SPAT, a bidirectional lncRNA, is located on human chromosome 12q24.33 (Supplementary Fig. S1B). Bioinformatics analysis revealed significantly lower expression of SPAT in LUAD tissues compared to normal lung tissues, a finding further validated in our clinical samples (Fig. 1B, C). We also examined SPAT expression across various human LUAD cell lines, discovering high expression levels in A549 and H460, and relatively lower levels in H1299, Hop62, H1650, H1975, and Calu1 (Supplementary Fig. S1C).

To assess the clinical significance of SPAT in LUAD, we analyzed the correlation between SPAT expression levels and patient clinical outcomes. Both overall survival and disease-free survival analyses indicated that high SPAT expression is associated with a favorable prognosis (Fig. 1D, E). Subcellular fractionation and RNA fluorescence in situ hybridization (FISH) analyses demonstrated that SPAT is predominantly localized in the nucleus (Fig. 1F and Supplementary Fig. S1D). Additionally, bioinformatic tools confirmed the noncoding nature of SPAT, as evidenced by low CPC score from both CPC2 and CPAT analyses (Fig. 1G).

### SPAT is a key regulator in the proliferation and migration of LUAD cells

**Fig. 2:**
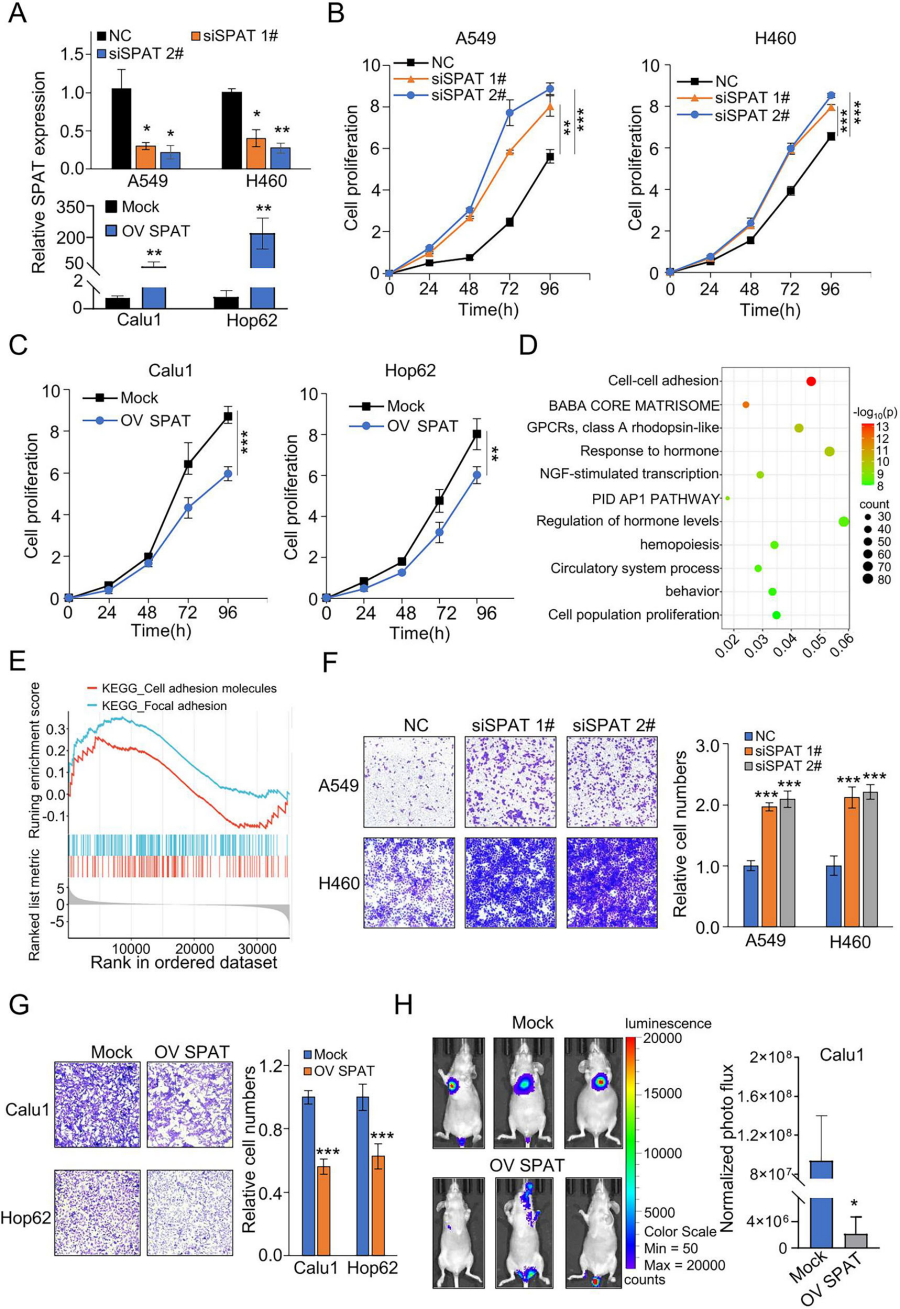
Altering SPAT expression has profound effects on proliferation and migration of LUAD cells. **A** qPCR analysis of SPAT expression in A549 and H460 cells transfected with SPAT siRNA or negative control (NC) siRNA, and in Calu1 and Hop62 cells transfected with SPAT overexpression or Mock vectors. **B** CCK-8 assays to assess cell proliferation in NC siRNA and SPAT knockdown LUAD cell lines. **C** CCK-8 assays to evaluate the proliferation of Calu1 and Hop62 cells transfected with SPAT overexpression or Mock vectors. **D** Enrichment analysis of signaling pathways and biological processes associated with differentially expressed genes upon SPAT knockdown. **E** GSEA enrichment plot analyzing adhesion-associated pathways based on RNA expression data from SPAT siRNA-transfected A549 cells and controls. **F**, **G** Transwell assays to assess the impact of SPAT knockdown and overexpression on the migratory capacity of LUAD cells. **H** Bioluminescence imaging and statistical results of metastatic lung colonization in nude mice intravenously injected with Mock or OV SPAT Calu1 cells via the lateral tail vein. Bioluminescence imaging was captured after 35 days.

To investigate the role of SPAT in LUAD cell proliferation, we generated SPAT knockdown cell lines in A549 and H460 cells, which exhibit high SPAT expression. The nuclear expression of SPAT was significantly downregulated by siRNA treatment. Additionally, we generated SPAT-overexpressing cell lines in Calu1 and Hop62 cells, which exhibit low endogenous SPAT expression levels (Fig. 2A and Supplementary Fig. S2A). CCK8 assays showed that SPAT knockdown significantly enhanced cell proliferation, a result corroborated with colony formation assays (Fig. 2B and Supplementary Fig. S2B). Conversely, SPAT overexpression markedly impeded LUAD cell growth (Fig. 2C and Supplementary Fig. S2C), indicating that SPAT plays an inhibitory role in LUAD cell proliferation. Moreover, we examined the effects of SPAT knockdown on the proliferation of LUAD primary cells, SIHA and KMH2 cells. Our results indicated that SPAT knockdown enhanced the proliferation of primary LUAD cells and SIHA cells but had no significant impact on the proliferation of KMH2 cells (Supplementary Fig. S2D–J).

Further RNA-seq analysis compared gene expression profiles between SPAT siRNA-treated and control siRNA groups (Supplementary Fig. S3A). Significantly altered genes upon SPAT knockdown were subjected to enrichment analysis, revealing cell-cell adhesion as the most relevant pathway (Fig. 2D). The SPAT-related co-expression network similarly highlighted adherens junction as a key enriched pathway (Supplementary Fig. S3B). GSEA analysis further revealed a negative correlation between SPAT expression and pathways related to cell adhesion and focal adhesion in LUAD cells, suggesting a potential role for SPAT in regulating LUAD cell migration and invasion (Fig. 2E).

To test this hypothesis, we performed transwell and scratch assays. As expected, SPAT depletion in A549 and H460 cells significantly enhanced migration and invasion, while SPAT overexpression inhibited these processes in Calu1 and Hop62 cells (Fig. 2F, G and Supplementary Fig. S3C–E). Western blot assays confirmed the upregulation of metastasis-associated protein TWIST following SPAT knockdown (Supplementary Fig. S3F). In SIHA and KMH2 cells, SPAT knockdown significantly promoted the migration of SIHA cells but had no observable effect on KMH2 cells (Supplementary Fig. S3G). To further assess SPAT’s functional involvement in LUAD metastasis, we intravenously injected stable SPAT-overexpression cells into the lateral tail veins of nude mice. Continuous bioluminescence imaging revealed a significant reduction in metastatic outgrowth in the lungs of mice injected with SPAT-overexpressing Calu1 cells over a period of 5 weeks (Fig. 2H and Supplementary Fig. S3H), Subsequent immunohistochemical (IHC) analyses of tumor tissues from nude mice revealed that SPAT overexpression decreased Ki67 and TWIST expression levels. Collectively, these findings strongly suggested that SPAT is a key regulator in LUAD cell proliferation and migration.

### SPAT interacts with SF1

**Fig. 3:**
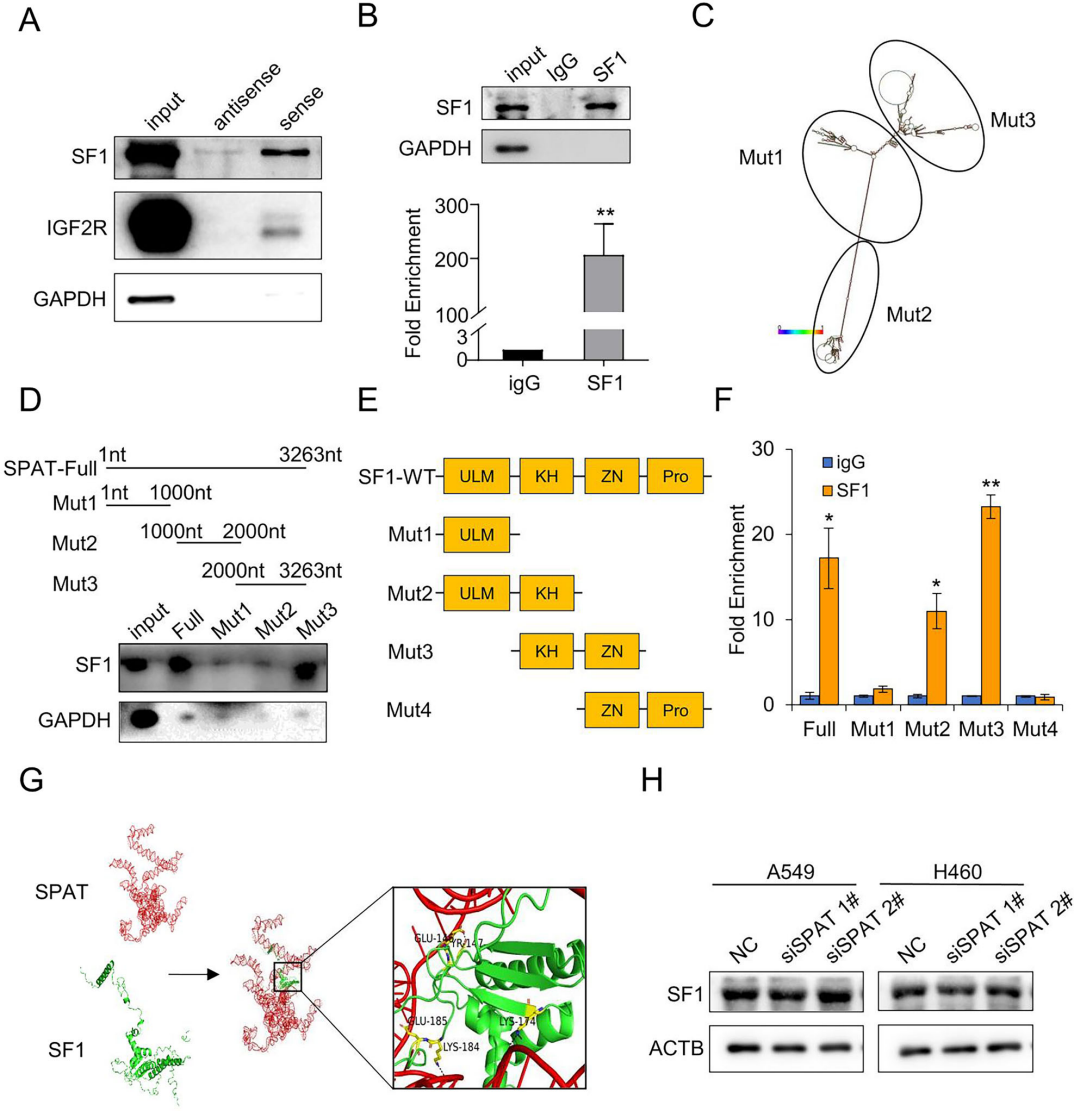
SPAT interacts with SF1. **A** Immunoblotting to validate the specific interaction of SPAT with SF1 and IGF2R, GAPDH serving as a negative control. **B** RIP assays demonstrating the association of SF1 with SPAT. Relative enrichment quantified RNA levels specifically associated with SF1 compared to an input control, with IgG as the negative control. **C** Schematic diagram of SPAT hairpin structures predicted by the RNAfold webserver. **D** Western blot analysis of full-length and truncated forms of SPAT in the pull-down fractions. GAPDH was used as a negative control. **E** Schematic diagram of SF1 and its truncated forms. **F** RIP assays using anti-FLAG antibodies to ascertain the enrichment of SPAT in various SF1 mutants. **G** Graphical representation of three-dimensional structures of SPAT and SF1 docking models, accompanied by a magnified image for enhanced visualization. Molecular docking sites between SPAT and SF1 include 2308 G/Glu146, 2329U/Tyr147, 2332U/Glu185, 2347 G/Lys174, and 2356 G/Lys184. **H** Western blot analysis of SF1 protein levels in SPAT knockdown and control cells.

To uncover the molecular mechanism of SPAT in LUAD, we performed RNA-pulldown followed by mass spectrometry to identify interacting proteins. SF1 emerged as one of the most abundant proteins co-precipitated with SPAT (Fig. 3A and Supplementary Fig. S4A, B). RNA immunoprecipitation (RIP) assays with an SF1 antibody further confirmed the interaction between SPAT and SF1 (Fig. 3B). To determine the specific region of SPAT that interacts with SF1, we utilized RNAfold to analyze the secondary structure of SPAT and identified three fragments for mutation analysis (Fig. 3C). Our results indicate that SF1 predominantly binds to a region spanning nucleotides 2000–3263 of SPAT (Fig. 3D).

SF1 contains four domains: ULM, KH, ZN, and PRO. Therefore, we generated truncation mutants of SF1 coupled with a FLAG tag plasmid and further confirmed that the KH domain is responsible for binding to SPAT (Fig. 3E, F). To further validate the interaction between the 2000-3263bp region of SPAT and the KH domain of SF1, we utilized 3dRNA to predict the tertiary conformation of the 2000-3263bp region in SPAT and obtained the corresponding 3D structure data for SF1 from Protein Data Bank [20]. Subsequently, HDOCK was employed to conduct in-silico molecular docking simulations between these two molecules [21]. Our results suggest potential hydrogen bond interactions within residues in both regions: specifically, within residues spanning nucleotides 2000-3263bp on SPAT and within the KH domain on SF1 (Fig. 3G).

Overall, our data confirmed that SPAT specifically interacts with SF1. We then explored the possibility of a regulatory connection between them. Interestingly, depleting SF1 showed no significant impact on SPAT levels, and likewise, disrupting SF1 did not noticeably affect SPAT either (Fig. 3H and Supplementary Fig. S4C).

### SPAT regulates alternative splicing of KITLG

**Fig. 4:**
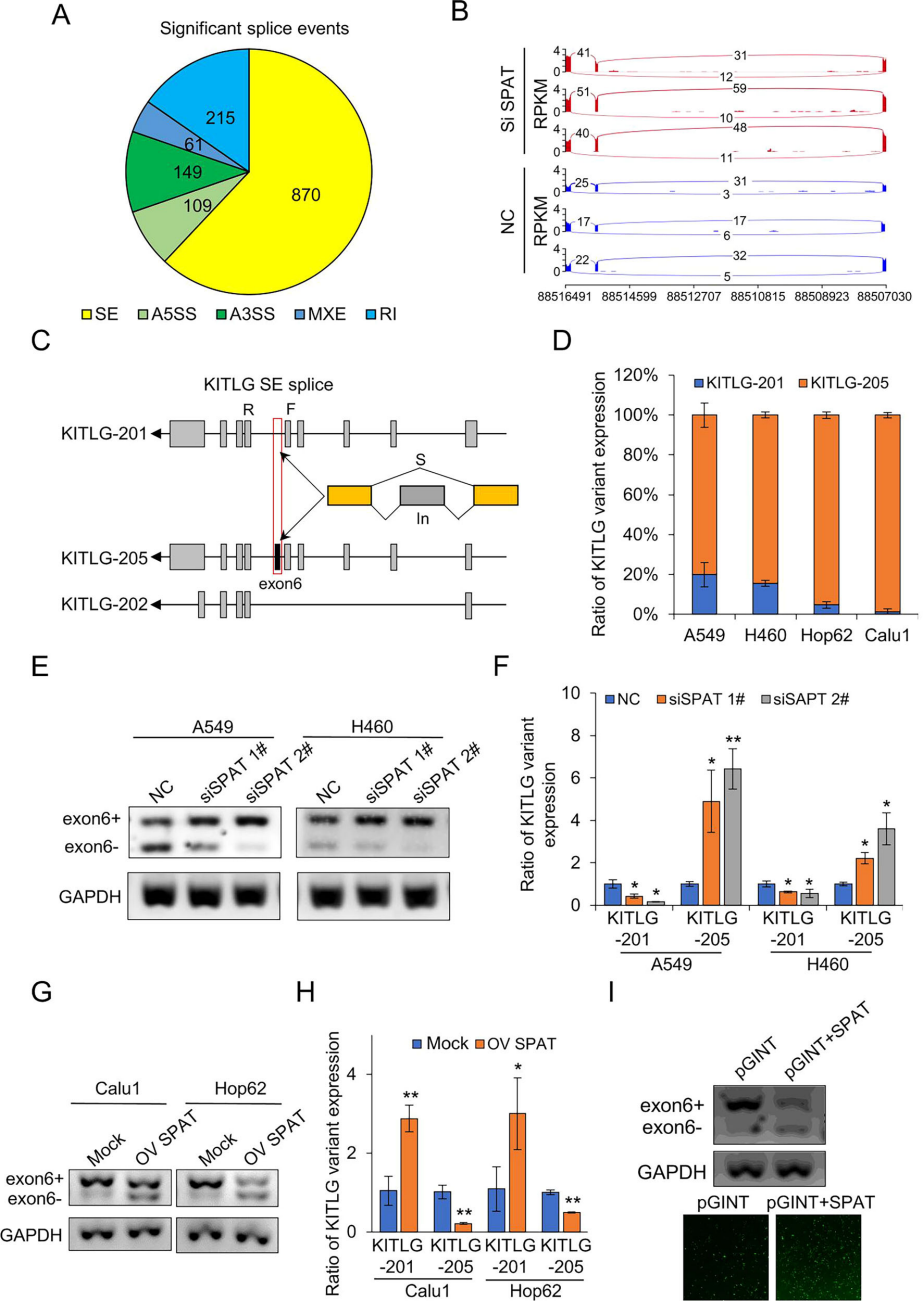
SPAT regulates alternative splicing of KITLG. **A** Splicing events, including SE (*n* = 870), RI (*n* = 215), A3SS (*n* = 149), A5SS (*n* = 109), and MXE (*n* = 61), observed in SPAT-knockdown A549 cells. **B** Sashimi plots visualizing the alternatively spliced transcripts of KITLG mRNA exon 6 in SPAT-knockdown A549 cells compared to control cells. **C** Schematic diagram illustrating KITLG exon 6+ (KITLG-205) and KITLG exon 6- (KITLG-201) isoforms. **D** qPCR analysis of the proportion of KITLG-205 and KITLG-201 transcripts in different LUAD cells using specific primers. **E**, **F** Semi-quantitative PCR and qPCR analysis of KITLG exon 6 AS events in SPAT-knockdown A549 and H460 cells. **G**, **H** Semi-quantitative PCR and qPCR analysis of KITLG exon 6 AS events in SPAT-overexpression Calu1 and Hop62 cells. **I** Semi-quantitative PCR analysis of KITLG exon 6 splicing in a pGint minigene reporter system in control or SPAT-overexpression A549 cells. Fluorescence signal of EGFP representing exon 6 exclusion efficiency.

Given the specific interaction between SPAT and SF1, and considering SF1’s crucial role in RNA splicing [22], we explored whether SPAT regulates SF1-mediated pre-RNA alternative splicing in LUAD. Using rMATS to analyze RNA-seq data, we identified significant changes in splicing events associated with metastasis in SPAT-knockdown A549 cells (Fig. 4A) [23]. Notably, SPAT knockdown led to a significant increase in the inclusion of KITLG exon 6, resulting in the generation of the KITLG-205 splice variant (Fig. 4B and Supplementary Fig. S5A). KITLG, also known as stem cell factor (SCF), is a pleiotropic factor encoding the ligand of the tyrosine-kinase receptor and plays crucial roles in cell migration, germ cell and neural cell development, and hematopoiesis [24]. This alternative splicing event involving exon 6 generates two transcripts: KITLG-201 (exon 6−) and KITLG-205 (exon 6+) (Fig. 4C). qPCR analysis using specific primers for these transcripts confirmed that endogenous expression of KITLG-205 was significantly higher than that of KITLG-201 (Fig. 4D).

Next, we conducted semi-quantitative RT-PCR to validate the alternative splice events using primers flanking the splice site responsible for generating KITLG-205 and KITLG-201. Our results unequivocally demonstrate that SPAT knockdown results in the downregulation of KITLG exon 6- and the upregulation of KITLG exon 6+ in LUAD cells (Fig. 4E). This observation was further confirmed by qPCR (Fig. 4F). Conversely, overexpression of SPAT attenuated the inclusion of KITLG exon 6+ while facilitating the skipping of KITLG exon 6-, as anticipated (Fig. 4G, H and Supplementary Fig. S5B, C). To gain deeper insights into this phenomenon, we established a KITLG exon 6 minigene reporter system by inserting exon 6 and its flanking intronic sequences into the pGint vector [25]. In this system, exclusion of exon 6 maintained the intact open reading frame (ORF) of EGFP, while inclusion of exon 6 disrupted the ORF, leading to reduced EGFP expression. Semi-quantitative PCR analysis confirmed that SPAT overexpression inhibited KITLG exon 6 inclusion, as evidenced by increased EGFP expression (exon 6 exclusion) in LUAD cells (Fig. 4I). To determine whether SPAT exerts a transcriptional role in modulating KITLG, we assessed KITLG pre-RNA levels in SPAT-knockdown A549 cells, but observed no significant changes (Supplementary Fig S5D). Collectively, these results suggest that SPAT primarily regulates the alternative splicing of KITLG, rather than its transcription.

### SPAT blocks SF1-mediated alternative splicing of KITLG

**Fig. 5:**
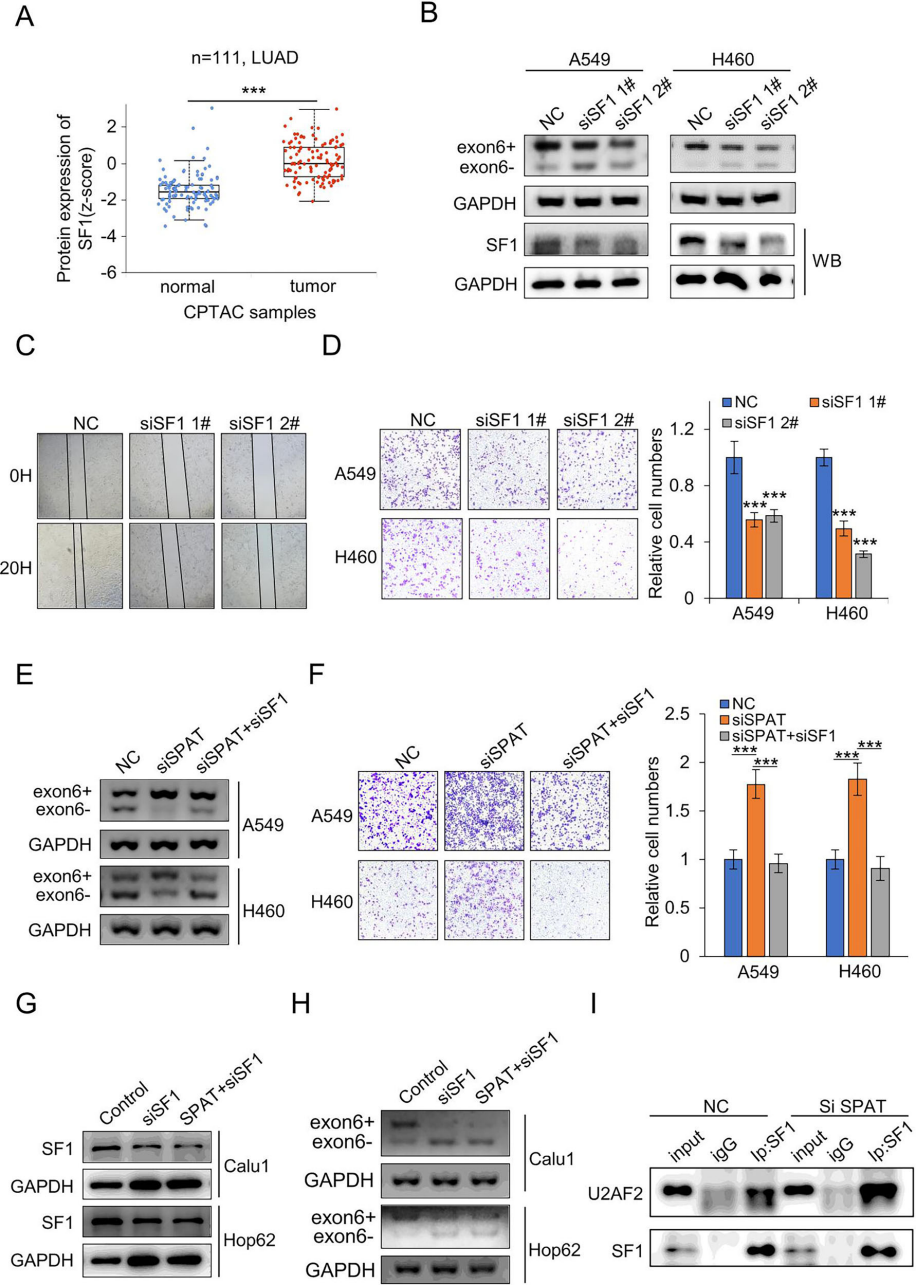
SPAT regulates alternative splicing of KITLG via block SF1. **A** Protein expression analysis of SF1 in LUAD tissues and adjacent tissues based on CPTAC samples. **B** Semi-quantitative PCR analysis of exon 6+ and exon 6- expression in SF1-knockdown A549 and H460 cells using a pair of primers. **C** Scratch test to detect the cell migration of A549 cells with SF1 knockdown after 20 h. **D** Transwell migration assays in SF1-silenced and control A549 and H460 cells. Representative images and statistical analysis are shown. **E** Semi-quantitative PCR analysis of KITLG exon 6 AS events in A549 and H460 cells transfected with NC, siSPAT, or siSPAT and siSF1 in combination. **F** Transwell migration assays in A549 and H460 cells co-transfected with siSPAT and siSF1. **G** Western blot analysis of SF1 protein levels in Calu1 and Hop62 cells co-transfected with siSF1 and SPAT overexpression vectors. **H** Analysis of KITLG exon 6 AS events in Calu1 and Hop62 cells. **I** Co-immunoprecipitation analysis of the interaction between SF1 and U2AF2 in NC and siSPAT groups.

Given the interaction between SPAT and SF1, along with SPAT’s impact on LUAD metastasis and KITLG splicing, we next investigated the role of SF1 in LUAD. Analysis of the CPTAC database (https://ualcan.path.uab.edu/index.html) revealed significant upregulation of SF1 protein levels in LUAD (Fig. 5A). Semi-quantitative RT-PCR and qPCR showed that SF1 knockdown in A549 and H460 cells resulted in reduced KITLG exon 6+ expression and increased KITLG exon 6- expression (Fig. 5B and Supplementary Fig. S6A). Conversely, SF1 overexpression had the opposing effect on KITLG splicing (Supplementary Fig. S6B). Scratch and transwell assays demonstrated that SF1 knockdown significantly impeded LUAD cell migration (Fig. 5C, D).

To address the role of SF1 in SPAT-mediated regulation of KITLG splicing, we downregulated SF1 in SPAT-deficient LUAD cells and observed a significant abrogation of SPAT-induced changes in KITLG exon 6 splicing and cell migration (Fig. 5E, F). To further elucidate whether SF1 is required for SPAT-mediated alternative splice events involving KITLG exon 6, we co-transfected LUAD cells with siRNAs targeting SF1 and SPAT. Semi-quantitative RT-PCR assays showed that SPAT’s influence on KITLG splicing was abolished in the absence of SF1, indicating that SPAT’s impact on KITLG splicing is SF1-dependent (Fig. 5G, H).

During early spliceosome assembly (complex E formation), SF1 recognizes the branch point sequence (BPS) and recruits U2 snRNP (including U2AF1 and U2AF2) to the spliceosome [26]. Co-immunoprecipitation assays revealed enhanced recruitment of U2AF2 by SF1 upon SPAT knockdown (Fig. 5I). We also explored the role of U2AF2 in splicing regulation. U2AF2 was found to be highly expressed in LUAD, and its knockdown resulted in a significant decrease in KITLG exon 6+ and an increase in KITLG exon 6-, accompanied by decreased LUAD cell migration (Supplementary Fig. S6C–G). Overall, these findings suggest that SPAT may exert its function by inhibiting SF1’s role in the alternative splicing of KITLG.

### Modulation of KITLG alternative splicing regulates the migration of LUAD cells

**Fig. 6:**
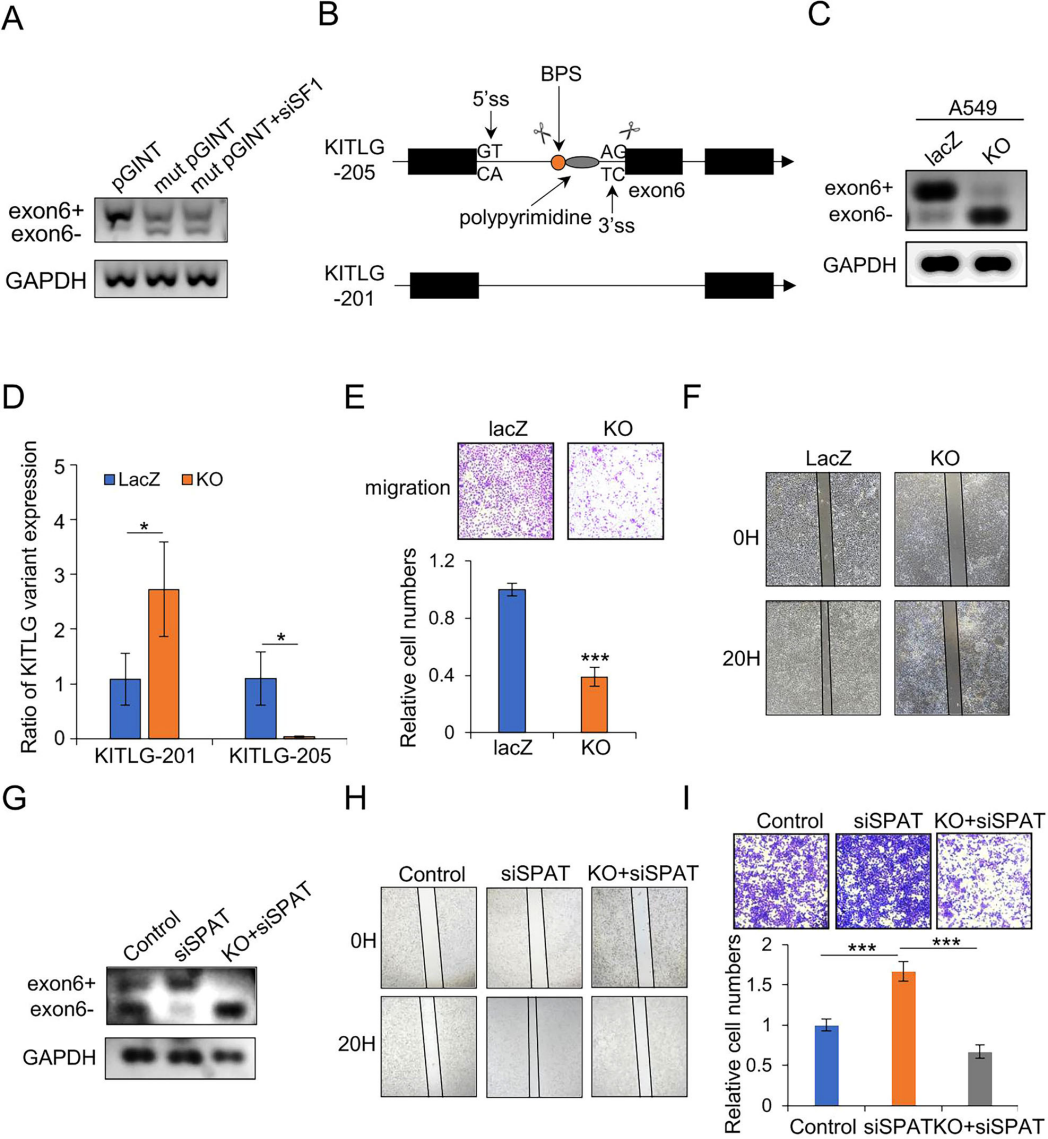
Modulation of KITLG splicing regulates the migration of LUAD cells. **A** Semi-quantitative PCR analysis of the impact of SF1 on KITLG exon 6 splicing after deletion of BPS. **B** Schematic diagram showing modulation of KITLG splicing. The scissor signifies the target of gRNA. **C**, **D** Semi- quantitative PCR and qPCR analysis of KITLG splicing in A549 cells with stable knockout of branch site and 3’SS site (KO). **E** Transwell assay to detect cell migration in the KO and LacZ control groups. **F** Wound healing assay to assess cell migration. **G** Analysis of AS in control, SPAT knockdown, and SPAT-deficient KO cells. **H**, **I** Transwell and scratch assays to assess the migration of A549 cells with various treatment.

SF1 recognizes the branch point sequence (BPS) during the initial stages of pre-mRNA splicing, which typically adheres to a YNCURAY consensus motif (Y = pyrimidine, N = any nucleotide, R = purine). BPSs are frequently located 15–45nt upstream of the 3’ splice site [27, 28]. Using SpliceAid2, we predicted the BPS motif (CACTGAC) recognized by SF1 during the alternative splicing of KITLG exon 6. Deletion of the BPS motif in a pGint minigene system confirmed that SF1 had no additional impact on the alternative splicing of KITLG exon 6, validating our prediction (Fig. 6A).

Given SPAT’s involvement in the alternative splicing of KITLG through SF1, we investigated whether modulation of KITLG splicing could affect the metastatic potential of LUAD cells. To induce exon 6 skipping in KITLG, we designed CRISPR sgRNAs targeting both the BPS site and 3’ splice site of exon 6 (Fig. 6B). These sgRNAs effectively induced exon 6 skipping (Fig. 6C, D). A549 cells expressing these sgRNAs exhibited reduced migration capacity, as observed in transwell and scratch assays (Fig. 6E, F). Rescue experiments further demonstrated that transient transfection of SPAT siRNA into A549 cells with knockout mutations at both the SF1 branch site and 3’ splice site restored alternative splicing, thereby reversing the migration induced by SPAT knockdown (Fig. 6G–I).

### Upregulated KITLG-205 facilitates the migration of LUAD cells

**Fig. 7:**
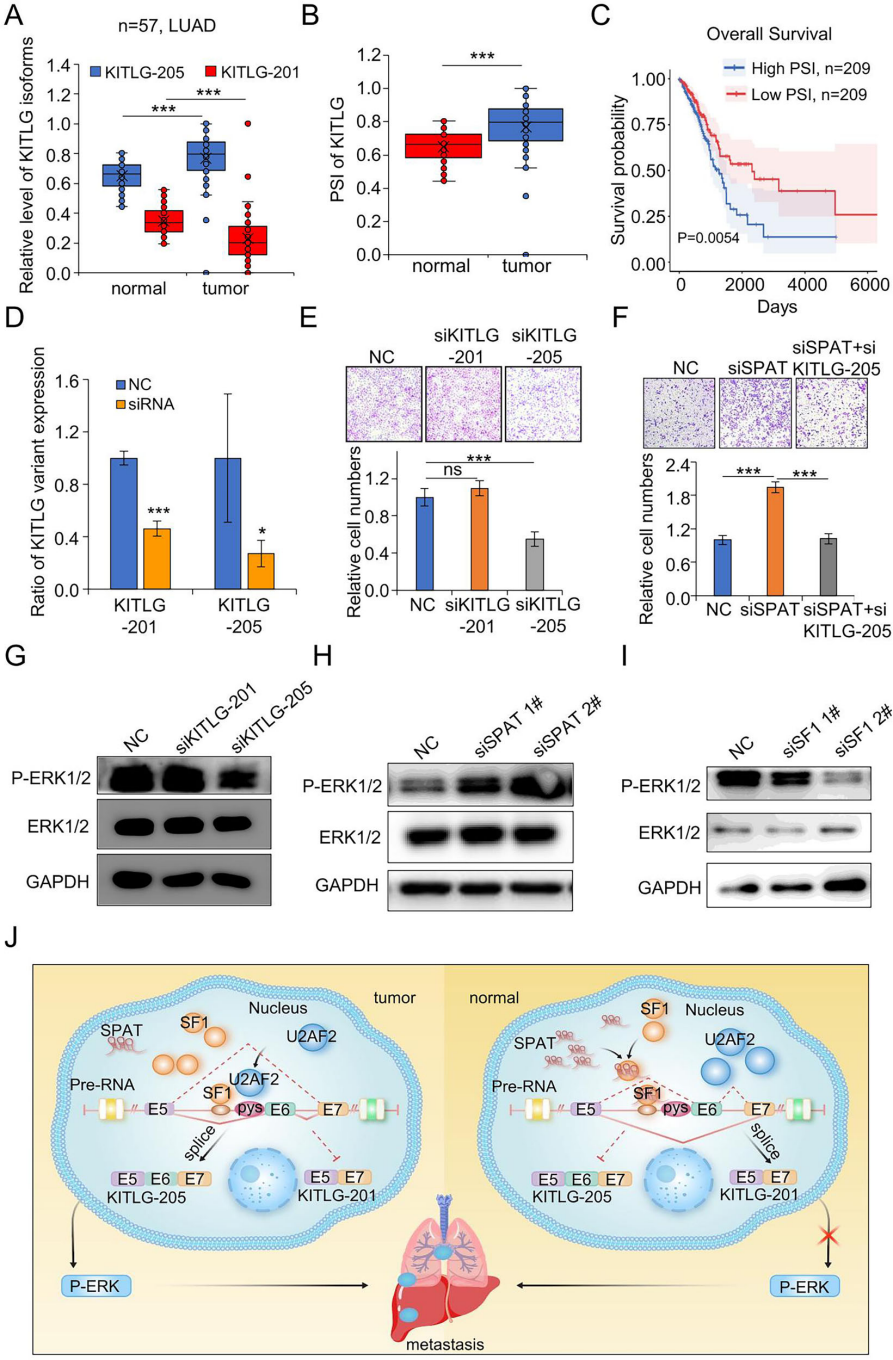
Upregulated KITLG-205 facilitates the migration of LUAD cells. **A** Relative expression of KITLG isoforms in clinical LUAD and matched-paired normal samples. **B** PSI of KITLG exon 6 in clinical LUAD and matched-paired normal tissues. **C** Kaplan–Meier plots of LUAD patients with high and low KITLG PSI. **D** qPCR analysis of the knockdown effect of siRNAs targeting KITLG-201 and KITLG-205 transcripts. **E**, **F** Transwell assay to assess the impact of silencing KITLG-201 and KITLG-205 transcripts on cell migration. **G** Western blot analysis of P-ERK1/2 levels after knockdown of KITLG-201 and KITLG-205 transcripts. **H** Western blot analysis of P-ERK1/2 levels in A549 cells transfected with NC and siSPAT. **I** Western blot analysis of P-ERK1/2 level in SF1 knockdown and control cells. **J** Proposed model of SPAT action in LUAD cell metastasis.

We analyzed the expression of two KITLG splicing variants in clinical LUAD tissues and observed a significant increase in KITLG exon 6+ variant expression, accompanied by a notable decrease KITLG exon 6- variant expression (Fig. 7A). However, there was no significant difference in the overall expression levels of KITLG (Supplementary Fig. S7A). The PSI (percentage splicing index) for KITLG exon 6 was much higher in LUAD samples than in matched normal tissues, and patients with lower PSI indices demonstrated improved survival outcomes (Fig. 7B, C).

Given SPAT’s involvement in LUAD metastasis and KITLG exon 6 splicing, we hypothesized that KITLG splicing variant might also contribute to LUAD metastasis. To investigate this, we designed specific siRNAs to target KITLG exon 6 inclusion (KITLG exon 6+) and KITLG exon 6 skipping (KITLG exon 6-) variants separately (Fig. 7D). Transwell assays revealed that knockdown of KITLG-201 did not exert a significant effect; however, suppression of KITLG-205 significantly attenuated the migratory capacity of LUAD cells and counteracted the migratory effect induced by SPAT knockdown (Fig. 7E, F).

Previous studies have demonstrated that KIT is activated by KITLG, leading to the activation of downstream AKT and extracellular signal-regulated kinase (ERK) signaling pathways [29]. We examined the effect of KITLG-205 and KITLG-201 isoforms on ERK activation. Interestingly, reduction of KITLG-205 decreased ERK phosphorylation, while reduction of KITG-201 had no effect on ERK phosphorylation (Fig. 7G). Furthermore, we found that SPAT knockdown upregulated ERK phosphorylation, whereas SF1 knockdown reduced ERK phosphorylation (Fig. 7H, I). These findings suggest that the upregulated KITLG-205 transcript enhances the migratory capacity of LUAD cells by modulating ERK phosphorylation.

## Discussion

The investigation of lncRNAs in LUAD has provided profound insights into their biological significance, yet the underlying mechanism by which lncRNAs contribute to LUAD pathogenesis remains inadequately elucidated [30]. In this study, we identified SPAT as a novel lncRNA implicated in LUAD through the construction of a co-expression network of lncRNAs. Notably, SPAT exhibits lower expression levels in LUAD compared to normal samples and is associated with a favorable prognosis for LUAD patients, suggesting its potential role as a tumor suppressor during LUAD progression.

Over recent decades, the field of lncRNA research has illuminated their pivotal roles in orchestrating intricate layers of gene expression program [31, 32]. Highly versatile and diverse lncRNAs create a supragenomic tier of gene regulation [33], mediated through variations in their sequences, structures, interaction partners, and subcellular localization patterns [34]. Given their pivotal influence on essential cellular processes, lncRNAs continue to be intensely studied for their contributions to development, physiology, and cancer progression [35]. Our functional analyses unveiled that depletion of SPAT promoted LUAD cell proliferation, invasion, and metastasis, whereas augmented expression of SPAT hindered these processes. Additionally, a murine model of lung cancer metastasis validated the inhibitory role of SPAT in LUAD cell metastasis.

Alternative splicing is regulated by a diverse array of splicing factors and alterations in splicing regulation are intricately linked to tumorigenesis [36]. Unraveling the mechanisms underlying aberrant splicing is crucial for understanding how the intricate machinery of splicing is governed and harmonized with other cellular processes, particularly transcription and signaling pathways [37]. Previous studies have highlighted lncRNAs’ involvement in alternative splicing and their contribution to cancer progression [38, 39]. For instance, LINC01089 collaborates with hnRNPM to promote HCC metastasis by modulating the splicing of invasion-related genes and activating the ERK signaling pathway [40]. Understanding splicing deregulation in cancer will lead to better comprehension of malignant transformation and may reveal new tools for cancer diagnosis, classification and innovative therapeutical interventions based on highly selective splicing correction approaches [41]. For example, alternative splicing can allow cancer cells to bypass germline BRCA1 mutations, contributing to therapeutic resistance [42]. Similarly, targeting specific splicing variants, such as CD44v5 in intrahepatic cholangiocarcinoma, has shown promising therapeutic potential [43].

Our study further revealed that SPAT directly interacts with SF1, which cooperates with U2AF2 to recognize the 3’ splice site during the initial stages of pre-mRNA splicing. This interaction suggests SPAT may be involved in alternative splicing of genes associated with metastasis. Subsequently, aberrant splicing of KITLG exon 6 was identified through rMATS analysis of RNA-seq data. Abnormal expression of KITLG and its receptor KIT has been reported in multiple epithelial-derived cancers, including breast, lung, and prostate cancers [44]. Our data demonstrated that SPAT regulates alternative splice of KITLG exon 6 by blocking splice activity of SF1. The absence of the BPS recognized by SF1 hinders exon 6 excision, effectively suppressing LUAD cell migration. Moreover, we observed a progressive increase in KITLG-205 expression while noting a declining trajectory for KITLG-201 in the LUAD cohort. Elevated PSI values were significantly associated with an unfavorable prognosis. Additionally, upregulated KITLG-205 promotes LUAD cell migration through ERK phosphorylation.

Currently, strategies targeting alternative splicing, including interventions aimed at SFs or the spliceosome, small molecules designed to modulate antisense oligonucleotides (SSOs), and RNA or protein isoforms, have demonstrated significant potential in clinical applications [45, 46]. Among these, H3B-8800, an oral small-molecule drug targeting SF3B1, has entered phase I clinical trials for the treatment of hematological malignancies [47]. SSOs, typically synthetic short-stranded RNAs, are engineered to base pair with cis-acting elements of precursor mRNA, thereby facilitating isoform switching by obstructing SF binding to precursor mRNA [48]. Our findings highlight the functional significance of SPAT in mediating LUAD progression via regulation of KITLG alternative splicing. Further exploration of AS modulation, specifically targeting SPAT or distinct KITLG isoforms, as a potential therapeutic strategy holds promise not only for LUAD but also for a broad range of cancer types.

In summary, our study demonstrates that SPAT regulates SF1-mediated alternative splicing events on KITLG exon 6, resulting in a decline in KITlG-205 transcripts and an elevation in KITlG-201 transcripts. The decline in KITLG-205 transcripts leads to reduced ERK phosphorylation, ultimately suppressing LUAD cell migration (Fig. 7J). Of therapeutic significance, the transcriptomic landscape of cancer cells renders them particularly susceptible to pharmacological inhibition of splicing. Emerging clinical trials with small-molecule splicing modulators and splice site-switching antisense oligonucleotides offer promising pathways for developing novel, personalized treatment strategies for LUAD [49].

## Methods

### Cell lines and cell culture

A549, H460, Calu1, HOP62, SIHA, KMH2 and HEK293T cell lines were purchased from ATCC (American Type Culture Collection, Manassas, VA, USA). LUAD primary cells were isolated according to a previously described protocol [50]. All cell lines were cultured in DMEM or RPMI 1640 medium (Gibco, Carlsbad, CA, USA) supplemented with 10% fetal bovine serum (FBS, Thermo Fisher Scientific, Rockford, IL, USA).

### Reagents and antibodies

Details regarding the reagents and antibodies utilized in this study are provided in Supplementary Table S1.

### siRNA and transfection

siRNAs were obtained from GenePharma (Shanghai, China) and transfected using the GenMute^TM^ reagent (SignaGen^TM^ Laboratories, Rockville, MD, USA). The specific siRNA target sequences are listed in Supplementary Table S2.

### RNA isolation and quantitative RT-PCR

RNA extraction was carried out using TRIzol RNA Isolation Reagents (Invitrogen, Carlsbad, CA, USA). Extracted RNA was reverse transcribed into cDNA using HiScript® II Reverse Transcriptase (Vazyme, Nanjing, China) according to the manufacturer’s instructions. Quantitative real-time PCR was performed using an SYBR Green PCR Mix Kit (Vazyme). The primers used in this study are listed in Supplementary Table S2.

### Plasmid construction and cell transfection

For the overexpression of SPAT, a full-length human SPAT sequence was synthesized by PCR and cloned into a PLVX-CMV-FLAG-Puro vector (Miaoling, Wuhan, China). To construct stably knockout SF1 + 3’SS splice bind site of KITLG exon 6 in A549 cell line, two sgRNAs were synthesized and cloned into the lentiCRISPRv2 vector (Addgene, Watertown, MA, USA). 293T cells were co-transfected with recombinant plasmids along with packaging plasmids pVSVG (Addgene) and psPAX2 (Addgene) for 48 hours to produce lentiviruses. A549 cells were then transfected with concentrated lentiviruses. Sequences of sgRNAs used are listed in Supplementary Table S2.

### Western blot

Total protein was isolated from cells using RIPA buffer (Beyotime) with a cocktail of proteinase inhibitors (PMSF, Beyotime). Proteins were quantified using a Pierce^TM^ BCA Protein Assay Kit (Thermo Scientific). Equal amounts of total protein were separated by 10% SDS–PAGE and transferred onto a PVDF membrane. Membranes were blocked with 5% skimmed milk and incubated the membranes with primary antibodies at 4 °C overnight, followed by incubation with secondary antibodies at room temperature for 2 h. Bands were detected using Pierce ECL Western Blotting Substrate reagent (Thermo Scientific).

### Immunohistochemistry

Fresh tissue samples were fixed in 4% paraformaldehyde, dehydrated through a graded alcohol series, cleared in xylene, embedded in paraffin, and sectioned at a thickness of 5–8 μM. Slides were deparaffinized in xylene, rehydrated through graded alcohols, and treated with 3% H₂O₂ in methanol for 10 min to block endogenous peroxidase activity. After rinsing in PBS, antigen retrieval was performed in citrate buffer at 95–100 °C for 10 min. Following cooling, the samples were blocked with serum, incubated with the primary antibody for 1 h, and washed. A biotinylated secondary antibody was applied for 30 min, followed by incubation with the streptavidin-HRP conjugate for another 30 min, protected from light. The signal was developed using DAB substrate, rinsed, counterstained with hematoxylin, dehydrated, cleared in xylene, and mounted. Staining was analyzed under a microscope.

### RNA fluorescence in-situ hybridization (FISH)

Approximately 6 × 10⁴ cells per well were seeded into a 24-well plate and cultured until they reached 80–90% confluency. SPAT-specific probes were synthesized by RiboBio (Guangzhou, China). Following fixation and permeabilization, cells were pre-hybridized at 37 °C for 30 min and then hybridized overnight with 50 μM SPAT probe in hybridization buffer at 37 °C. Coverslips were washed three times with wash buffer I and once each with wash buffers II and III, as well as PBS, at 42 °C, according to the RNA FISH protocol (GeneBio). After staining with DAPI for 10 min, coverslips were rinsed three times in PBS. Representative images of RNA FISH were obtained using confocal microscopy.

### Cell proliferation and colony assays

To assess cell variability, 24 h after transfection with siRNAs or plasmids, 1000 cells were seeded into 96-well plates. Cell proliferation was assessed using the Cell Counting Kit-8 (MCE, Monmouth Junction, NJ, USA). For colony formation assays, 1000 transfected cells were seeded in 12-well plates and then allowed to ~10 days. Colonies were fixed with methanol, stained with 0.1% crystal violet, and washed with PBS.

### Cell migration and invasion assays

Cells were diluted into a density of 1 × 10^5^ using RPMI-1640 medium without FBS. For cell migration, 300 μL of diluted cells were added into transwell chambers (Corning, Costar, Tewksbury, MA, USA) placed in a 24-well plate. For cell invasion, 300 μL of diluted cells were added into transwell chambers pre-coated with Matrigel (Corning). A total of 600 μL of RPMI-1640 medium supplemented with 10% FBS was added into the 24-well plates and cultured for 20 h. The cells on the inside of the chamber membranes were removed, fixed with methanol, and stained with 0.1% crystal violet. Cells underside of the membrane was then counted and imaged under a microscope (Leica DM4000, Buffalo Grove, IL, USA).

### Wound healing assays

Cells were seeded in six-well plates and cultured in a medium containing 10% FBS. Once the monolayer cells achieved 90–100% confluence, a vertical scratch wound was created using a 200-µL pipette tip. PBS was employed to wash away the floating cells, and the remaining cells were cultured in serum-free medium. Photographs were captured at 0 and 20 h post-scratching.

### RNA pull-down

RNA pull-down assays were performed as previously described [51]. Briefly, Biotin-labeled SPAT RNAs, including sense and antisense RNAs, were obtained using Ribo™ T7 transcription Kit (C11002-1), Fifty pmol of biotin labeled probes were incubated with streptavidin beads for 30 min at room temperature. Cell lysate from A549 cells were then added to the RNA-beads complex and incubated overnight at 4 °C. Subsequently, the proteins were eluted from the beads and separated by 10% SDS-PAGE followed by silver staining.

### RNA immunoprecipitation

RIP assays were performed using a Magna RIP RNA-binding Protein Immunoprecipitation Kit (Millipore) according to the manufacturer’s instruction. Briefly, cell lysates prepared in hypotonic buffer supplemented with RNase inhibitor and protease inhibitor were incubated with magnetic beads pre-incubated with SF1 antibodies at 4 °C overnight. After being washed with RIP wash buffer, the bead-bound immunocomplexes were subjected to immunoblotting analysis and RNA isolation. The RNA precipitated by RIP was detected by reverse transcription PCR and qPCR.

### RNA-seq and data analysis

Total RNAs isolated from three independent groups of A549 cells transfected with SPAT siRNA, or control siRNA were used for RNA-seq. RNA libraries were prepared for sequencing using HiSeq X10 sequencer (Mingma, Shanghai, China). Sequence reads were trimmed for adaptor sequence, and then mapped to human reference genome (hg38) using TopHat2 (v2.0.13). Gene expression was quantified using FPKM.

### Analysis of aberrant splicing of KITLG

To identify different AS events resulting from SPAT knockdown, we performed rMATS using RNA-Seq data. PSI was calculated as follows: PSI = (I /LI)/(I/LI + S/LS), where PSI represents the inclusion level, I is the inclusion junction counts, LI is the length of inclusion form, S is the skipping inclusion junction counts, and LS is the length of skipping form.

### Animal experiments

To determine the effect of SPAT on metastasis, 2 × 10^6^ Calu1 cells stably expressing SPAT and control cells were injected intravenously into the tail vein of 6-week-old female BALB/c nude mice. Metastasis was monitored by luciferase imaging of live animals using an IVIS@ Lumina II system (Caliper Life Sciences, Hopkinton, MA, USA), once a week for 5 weeks. All experiments were performed in accordance with the Guide for the Care and Use of Laboratory Animals (NIH publication 80-23, revised 1996), with approval of Zhejiang University (Approval No. ZJU202100112), Hangzhou, China.

### Tissue specimens

A total of 25 LUAD samples were obtained from the tissue bank of Sir Run Run Shaw Hospital of Zhejiang University. All tissue samples used in this study were obtained with permission at the time of diagnosis before any treatment was administered. The study protocol was approved by the Institutional Review Boards of Sir Run Run Shaw Hospital of Zhejiang University.

### Statistical analysis

Data are presented as mean ± SEM of at least three independent experiments. Statistical analyses were performed with the Student’s *t* test, with *p* value < 0.05 considered significant. Statistical significance is represented in figures by: 0.01 < ^*^P < 0.05; 0.001 < ^**^P < 0.01; ^***^P < 0.001.

## Supplementary information

Suppl Tables S1-S4 and Figures S1-S7

Original data

## Data Availability

This published article and its supplementary files contain all the data generated during this study.
