# Supplementary material for: SPAT inhibits LUAD metastasis by targeting SF1-mediated splicing
Source: Cell Death Dis. 2025 Aug 8;16(1):598. doi: 10.1038/s41419-025-07924-2 (PMC12334704; doi:10.1038/s41419-025-07924-2)
Supplement: Supplementary file 1 — Suppl Tables S1-S4 and Figures S1-S7 [file 41419_2025_7924_MOESM1_ESM.pdf]

Supplemental information

SPAT Inhibits LUAD metastasis by Targeting SF1-Mediated Splicing

Yun Ma[1,†], Xiaoxu Zhou[2,†], Mengqian Yu[2,†], Xiang Cheng[2,†], Juze Yang[1], Jiayi Ren[1], Chengcai Zheng[2], Jia Li[1], Xinyi Qian[1], Jiani Yi[1], Honghe Zhang[3], Yan Lu[2,*], Pengyuan Liu[1,4*].

Table S1. List of reagents and antibodies used in this study

Name	Manufacturer	Cat. no.
D-Luciferin	Beyotime	ST196-500mg
GAPDH	Proteintech Group	60004-1-Ig
TWIST1	Cell Signaling Technology	46702s
SF1	Abclonal	A6424
FLAG	Sigma-Aldrich	F1804
ACTB	Abclonal	AC026
U2AF2	Abclonal	A4552
Ki67	Cell Signaling Technology	9129
ERK1/2	Cell Signaling Technology	9102
Phospho-ERK1/2	Cell Signaling Technology	9101

Table S2. Sequences of primers, sgRNAs and siRNA used in the study

Oligo name	Sequence (5'→3')
SPAT-F	TGTATTAGTCCATTTTCACGCTGCTG
SPAT-R	AGATAACTTGTTTTGGCTATGTCCCC
GAPDH-F	AGAAGGCTGGGGCTCATTTG
GAPDH-R	AGGGGCCATCCACAGTCTTC
Actin-F	GGACTTCGAGCAAGAGATGG
Actin-R	AGCACTGTGTTGGCGTACAG
NEAT1-F	CCAGTTTTCCGAGAACCAAA
NEAT1-R	ATGCTGATCTGCTGCGTATG
KITLG Exon6-F	AACCCAGGCTCTTTACTCCTG
KITLG Exon6-R	ACTTGGCTGTCTCTTCTTCCA
KITLG-201-F	GCTTCGCCCTCTGCGTCCCCG
KITLG-201-R	CAGTTCCCGCCCGCCTGCTCC
KITLG-205-F	TCCAGAGTCAGTGTCACAAAA
KITLG-205-R	CCAGTATAAGGCTCCAAAAGC
KITLG (pre-mRNA)-F	GCAGATATCAATAGCACAGAAGA
KITLG (pre-mRNA)-R	TAATACAGAGTAACTGGGGAGGA
pGINT (KITLG Exon6)-F	AGCAAGGGCGAGGAGCTG
pGINT (KITLG Exon6)-R	GGTCTTGTAGTTGCCGTCGTC
Negative Control siRNA	UUCUCCGAACGUGUCACGUTT
SPAT siRNA1	GUGCCACUUUAGAUUAUAUTT
SPAT siRNA2	GCUAUGUAUACAAGACUUATT
SF1 siRNA1	CAGAGAUGGUUGCACUCAATT
SF1 siRNA2	GACGAUAACAGGAUCUUAATT
U2AF2 siRNA1	GCUCAACGAGAAUAAACAATT
U2AF2 siRNA2	ACAGAGUGGUUGUCACAAATT
KITLG-201 siRNA	GCGAGGUAUUUCGUCUGUCTT
KITLG-205 siRNA	CCAGAGUCAGUGUCACAAATT
lacZ sgRNA	CACCGTGCGAATACGCCCACGCGAT
BPS sgRNA1	AATAATGTGTCAGTGTTATA
BPS sgRNA2	TGGTTTTGTGACACTGACTC

Table S3. Quality control metrics of the RNA-seq libraries

Sample ID	Yield (Gb)	% of>= Q30 Bases	Mean Quality Score	Mapping rate (%)
NC-1	15.0	97.9	35.3	96.9
NC-2	19.8	98.2	35.4	96.7
NC-3	15.2	97.8	35.2	97.0
siSPAT-1	18.2	97.9	35.3	97.1
siSPAT-2	17.2	98.1	35.4	97.3
siSPAT-3	19.0	98.1	35.4	97.1

Table S4. Proteins identified by mass spectrometry analysis of RNA pull-down fractions.

Protein name	Unique peptides		Mol. Weight [kDa]
	Antisense	Sense	
IGF2R	0	10	274.2
**SF1**	**0**	**8**	**68.3**
IPO5	0	7	123.6
TPR	0	7	267.1
PTGR1	0	6	35.8

Note: Proteins that were only present in the sense fraction and had multiple detected peptides are preferentially verified by subsequent western blot and RIP assays.

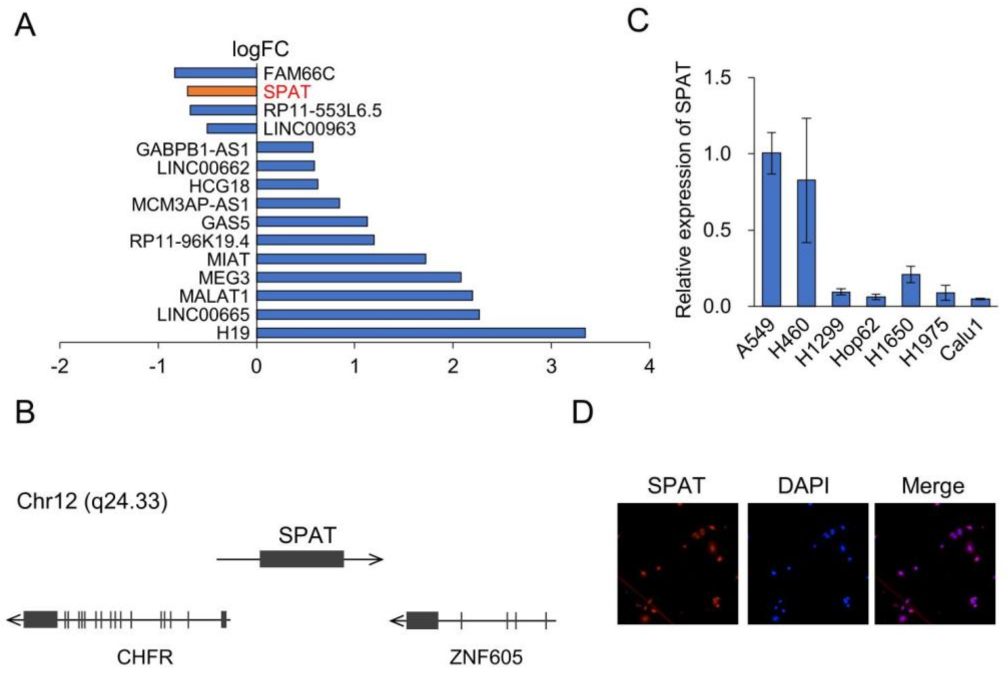

Figure S1. Related to Figure 1.

(**A**) Relative expression levels of individual lncRNAs that were identified by WMDS.net within the mRNA-lncRNA co-expression network were assessed in LUAD. Negative scores indicate reduced gene expression levels in tumors compared to normal tissues, whereas positive scores indicate increased gene expression levels in tumors compared to normal tissues.

(**B**) Schematic representation depicting the genomic location of SPAT and its neighboring genes, with arrows indicating the direction of transcription.

(**C**) Expression levels of SPAT in various LUAD cell lines.

(**D**) RNA fluorescence in-situ hybridization of SPAT in A549 cell.

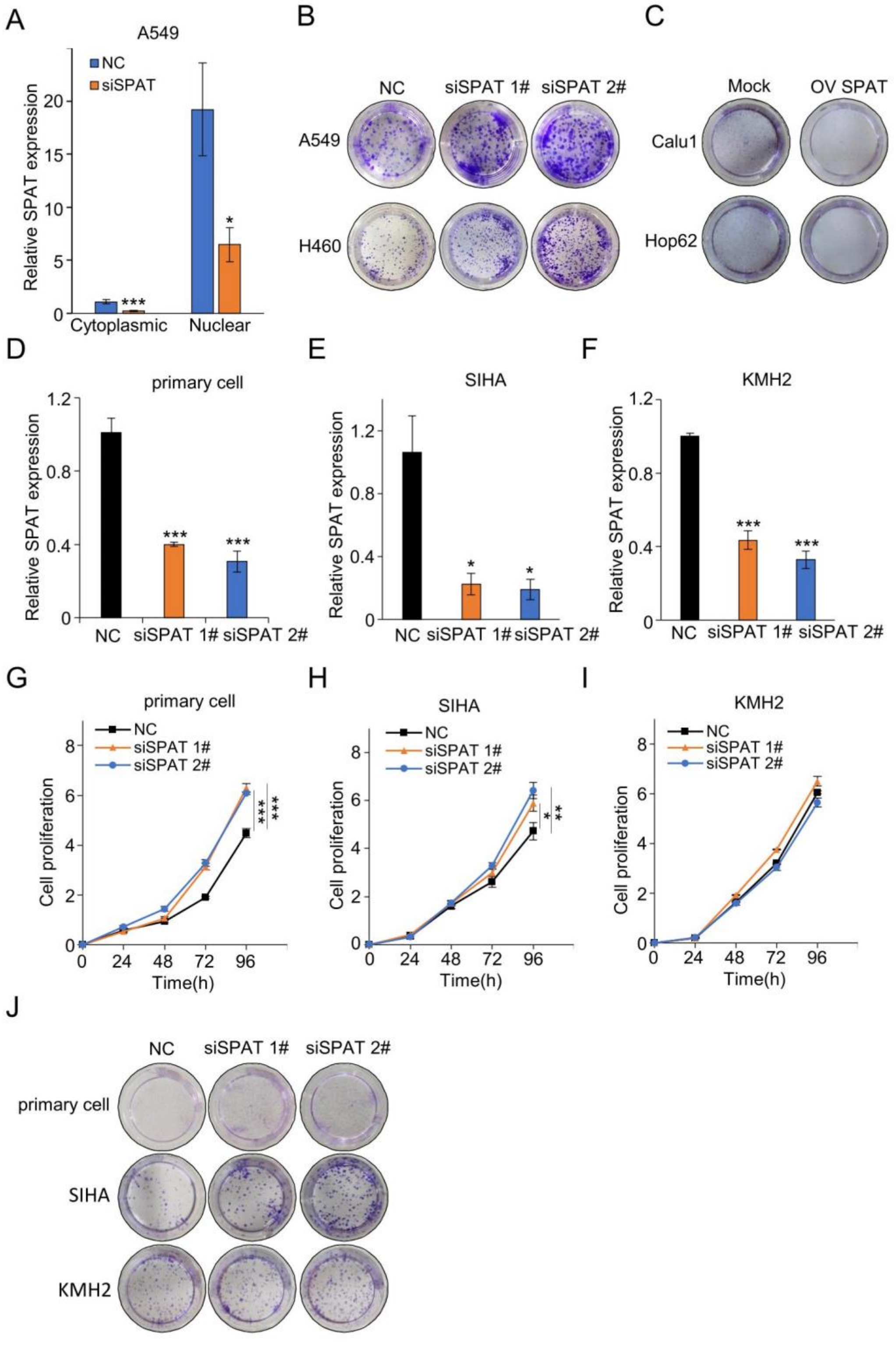

Figure S2. Related to Figure 2.

(**A**) qPCR analysis of the efficiency of SPAT siRNA knockdown in the nucleus and cytoplasm.

(**B, C**) Colony formation assays to assess the impact of SPAT on the viability of LUAD cells.

(**D, E, F**) qPCR analysis of SPAT expression in LUAD primary cell, SIHA and KMH2 cells transfected with SPAT siRNA or negative control (NC) siRNA.

(**G, H, I**) CCK-8 assays to assess cell proliferation in NC siRNA and SPAT knockdown indicated cells.
(**J**) Colony formation assays to assess the effect of SPAT knockdown on the proliferation ability of indicated cells.

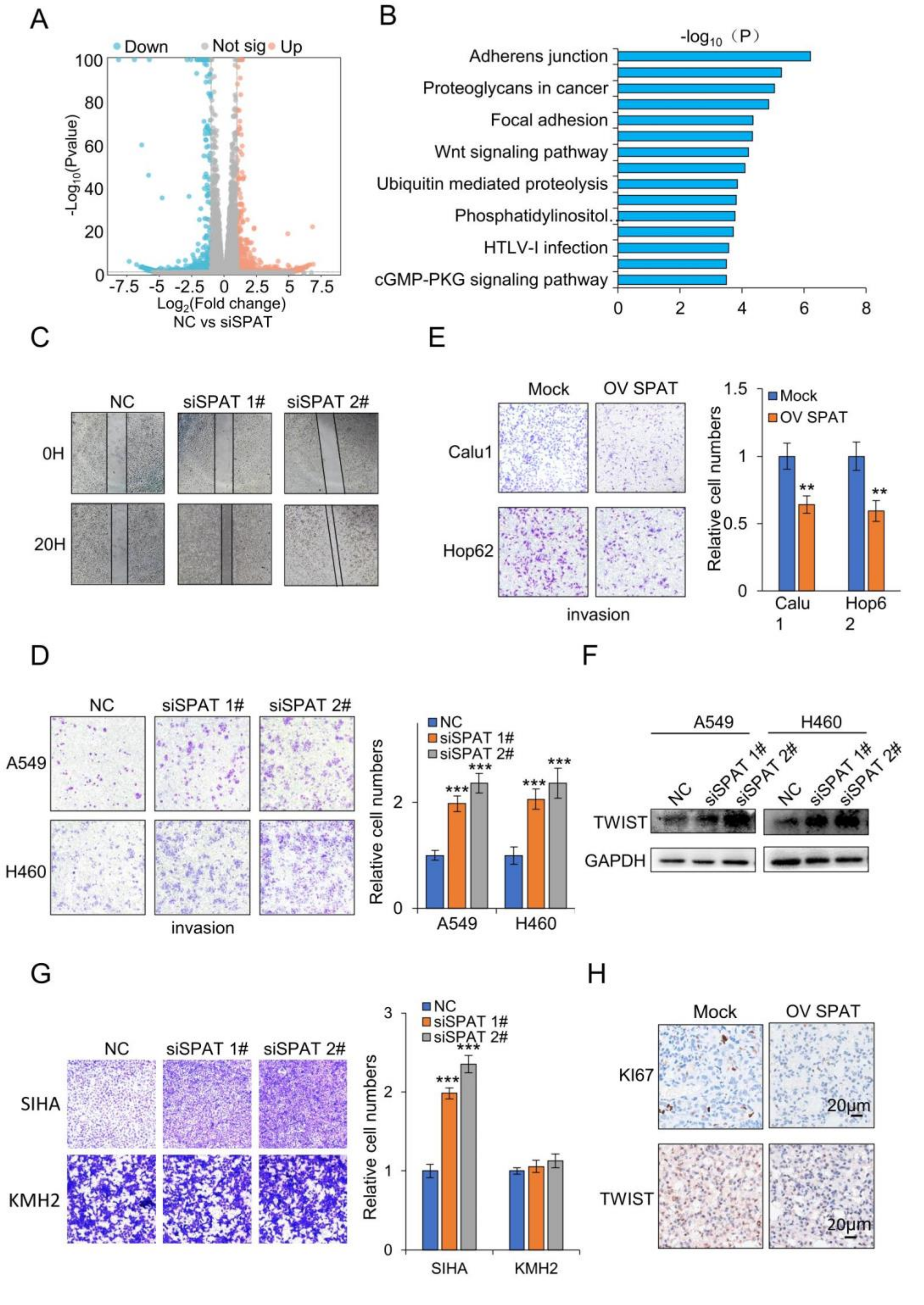

Figure S3. Related to Figure 2.

(**A**) Volcano plot illustrating differential gene expression following SPAT knockdown, (|log2 FC| >1, and $P < 0.05$ from two-sided Student's t-test).

(**B**) Enrichment analysis of SPAT-related genes derived from the mRNA-lncRNA co-expression network.

(**C**) Scratch assay to assess the impact of SPAT knockdown on the migratory capacity of A549 cells.

(**D, E**) Transwell assays to assess the invasion ability of A549 and H460 cells following SPAT knockdown and overexpression.
(**F**) Western blot analysis of TWIST protein levels in SPAT knockdown and control cells.
(**G**) Transwell assays to assess the impact of SPAT knockdown on the migratory capacity of SIHA and KMH2 cells.
(**H**) Representative images of immunohistochemical staining for KI67 and TWIST in lung tissues of nude mice intravenously injected with Mock or OV SPAT Calu1 cells.

A

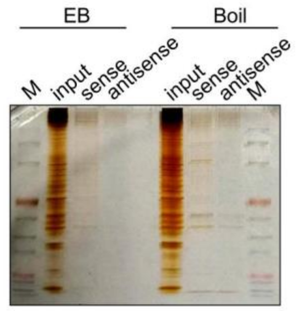

B

Gene Symbol	Unique peptides	Description
IGF2R	10	insulin like growth factor 2 receptor
SF1	8	Splicing factor 1
IPO5	7	Nucleocytoplasmic transport
TPR	7	nuclear basket protein
PTGR1	6	chemotactic factor

C

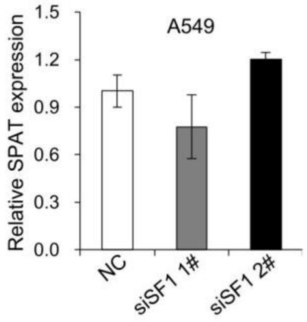

Figure S4. Related to Figure 3.
(**A**) Identification of proteins interacting with SPAT through a pull-down assay followed by mass spectrometry.
(**B**) Proteins identified by mass spectrometry analysis of RNA pull-down.
(**C**) qPCR analysis of SPAT expression in A549 cells transfected with siSF1 or NC.

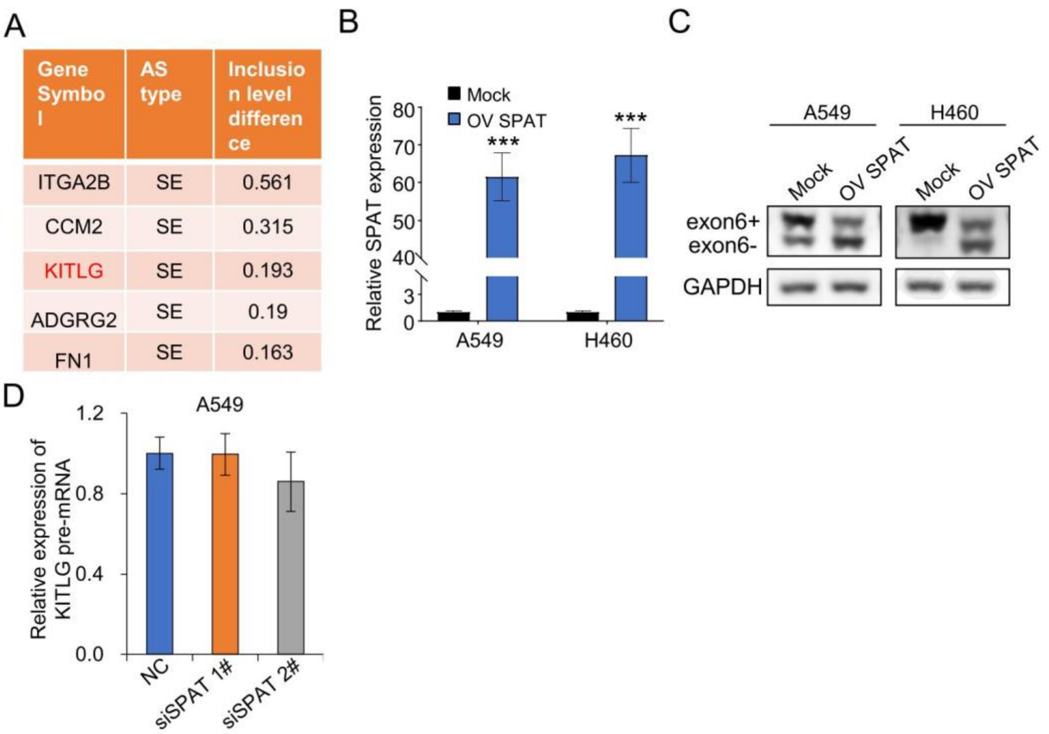

Figure S5. Related to Figure 4.

(**A**) Pronounced modulation in the alternative splicing of metastasis-associated genes following SPAT knockdown.

(**B**) qPCR analysis of SPAT overexpression in A549 and H460 cells.

(**C**) Semi-quantitative PCR analysis of KITLG exon 6 AS events after SPAT overexpression in A549 and H460 cells.

(**D**) qPCR analysis of KITLG pre-RNA in SPAT-knockdown and control A549 cells.

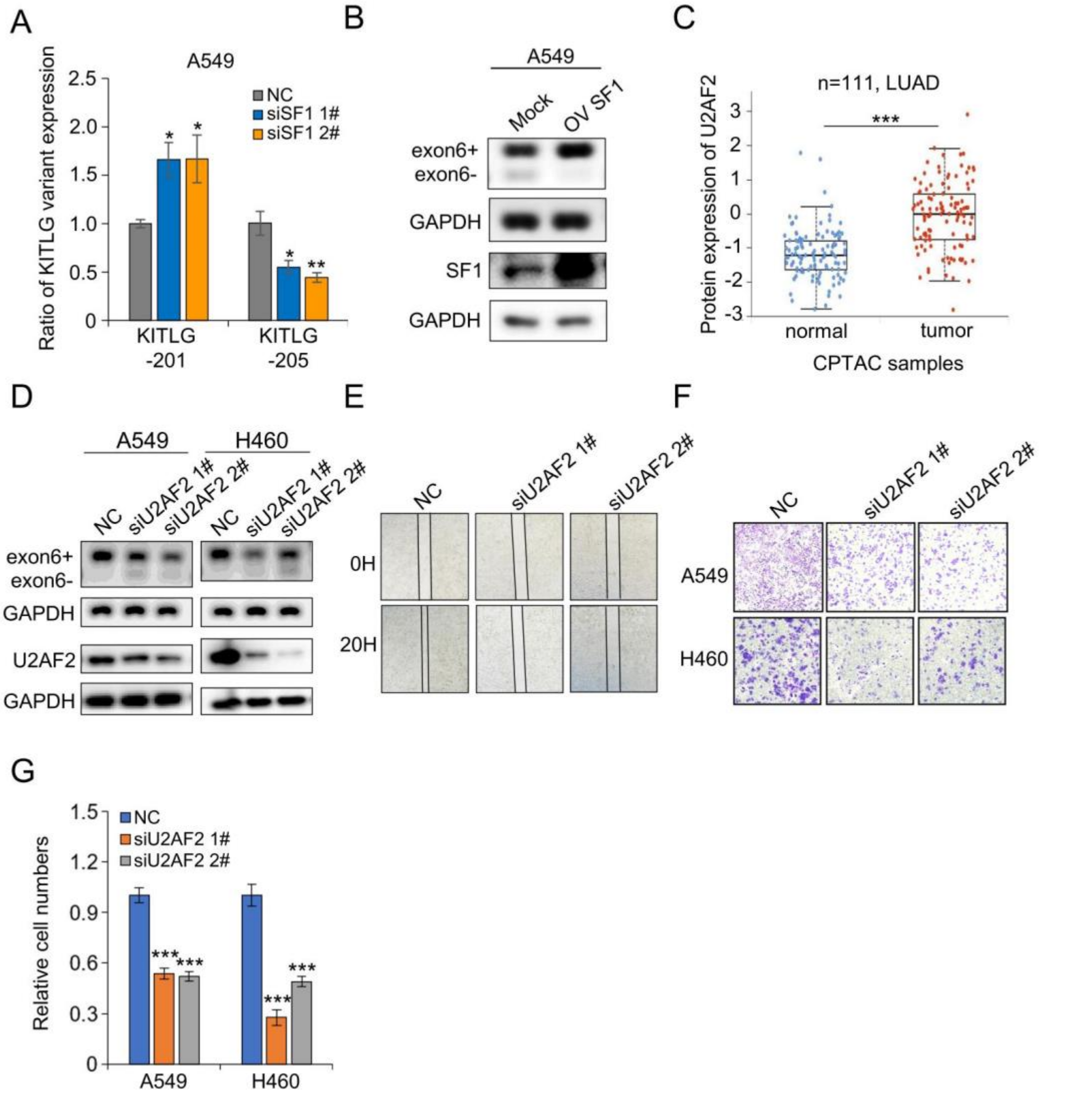

Figure S6. Related to Figure 5.

(**A**) qPCR analysis of the impact of SF1 knockdown on KITLG-201 and KITLG-205 transcripts.

(**B**) Semi-quantitative PCR analysis of KITLG exon 6 AS events following SF1 overexpression in A549 cells.

(**C**) Protein expression levels of U2AF2 in LUAD tissues and adjacent tissues based on CPTAC samples.

(**D**) Semi-quantitative PCR analysis of KITLG exon 6 AS events in control or U2AF2-knockdown A549 and H460 cells.

(**E**) Wound healing assay to evaluate the migratory behavior upon U2AF2 knockdown in A549 cells.

(**F, G**) Transwell migration assay to evaluate the migratory potential of A549 and H460 cells transfected with NC or siU2AF2.

A

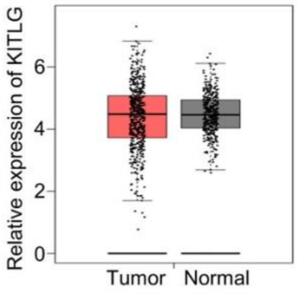

Figure S7. Related to Figure 7.
(**A**) Relative expression of KITLG in LUAD tissues and adjacent normal tissues in the TCGA cohort assessed by GEPIA.
