# Supplementary material for: SPAT inhibits LUAD metastasis by targeting SF1-mediated splicing
Source: Cell Death Dis. 2025 Aug 8;16(1):598. doi: 10.1038/s41419-025-07924-2 (PMC12334704; doi:10.1038/s41419-025-07924-2)

Supplementary Material: Original Uncropped Western Blots

**FIG S3**


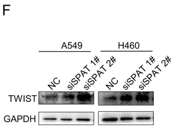


TWIST


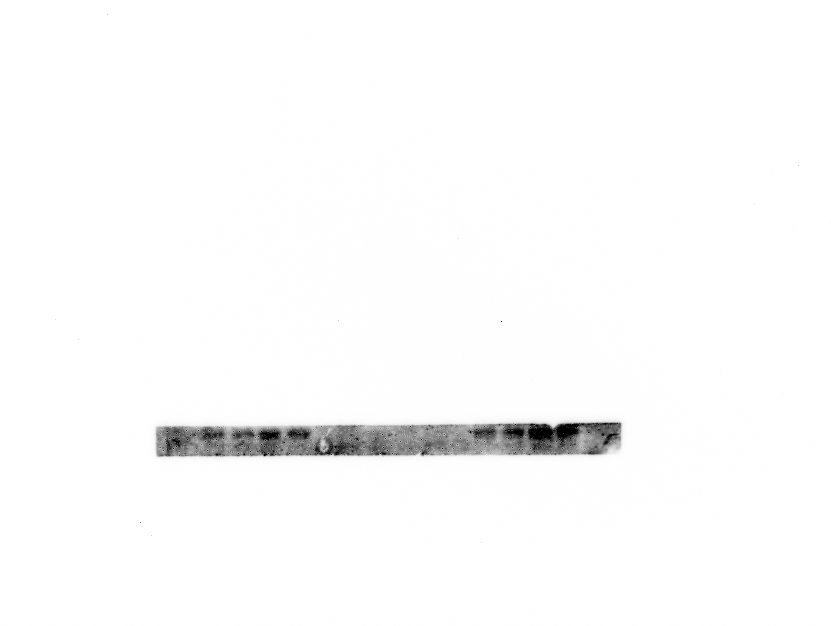


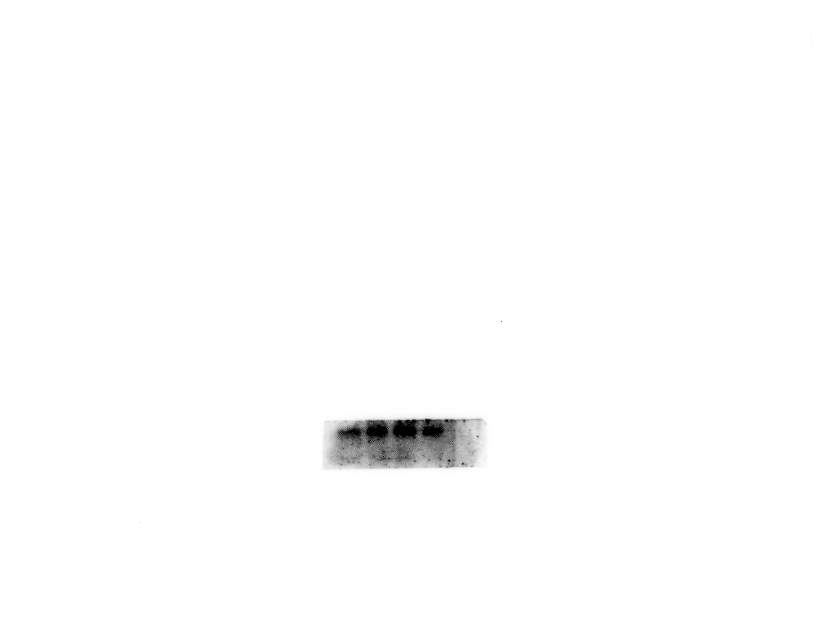


GAPDH


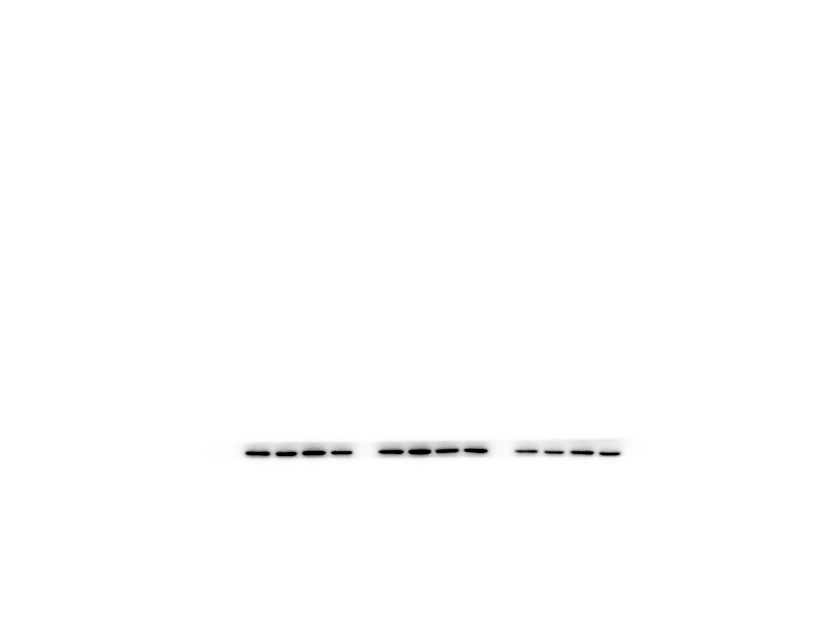


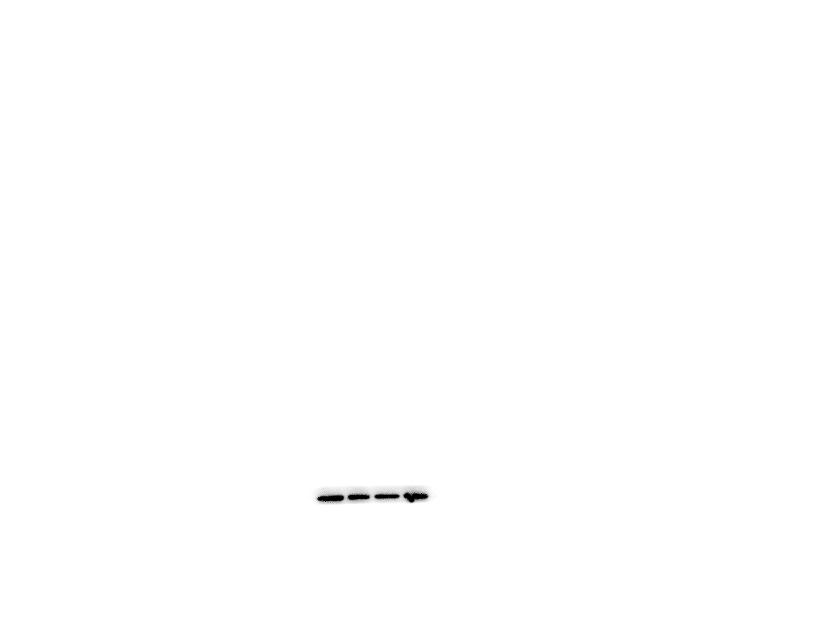


**FIG 3**


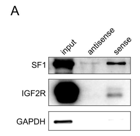


SF1


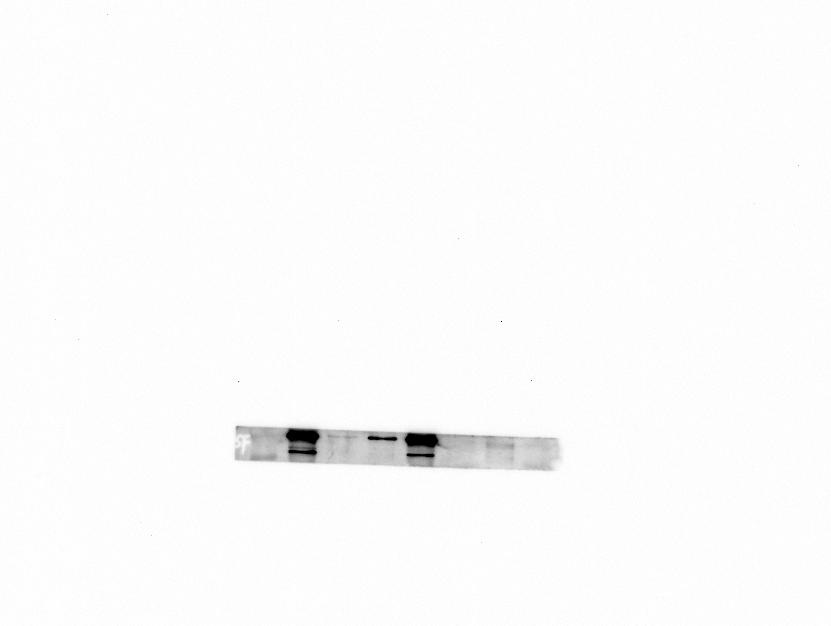


IGF2R


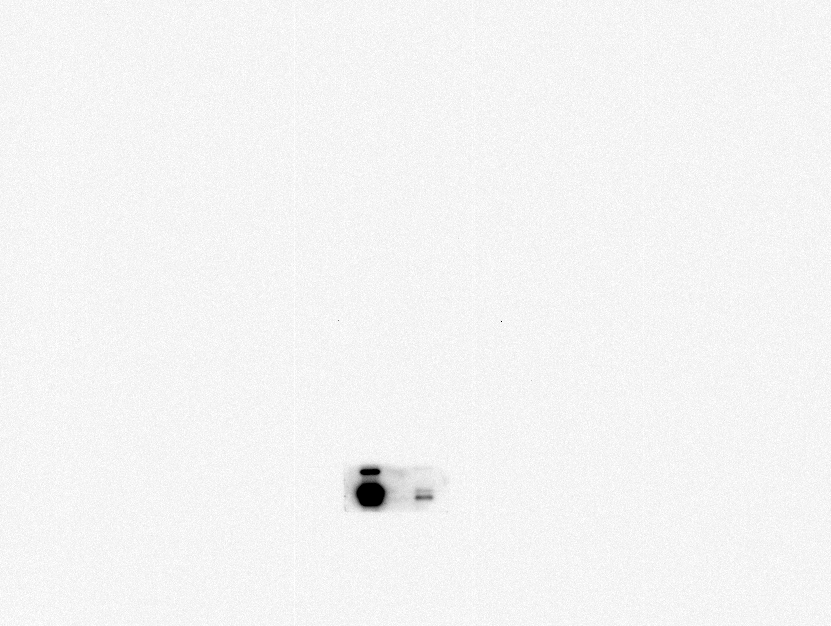


GAPDH


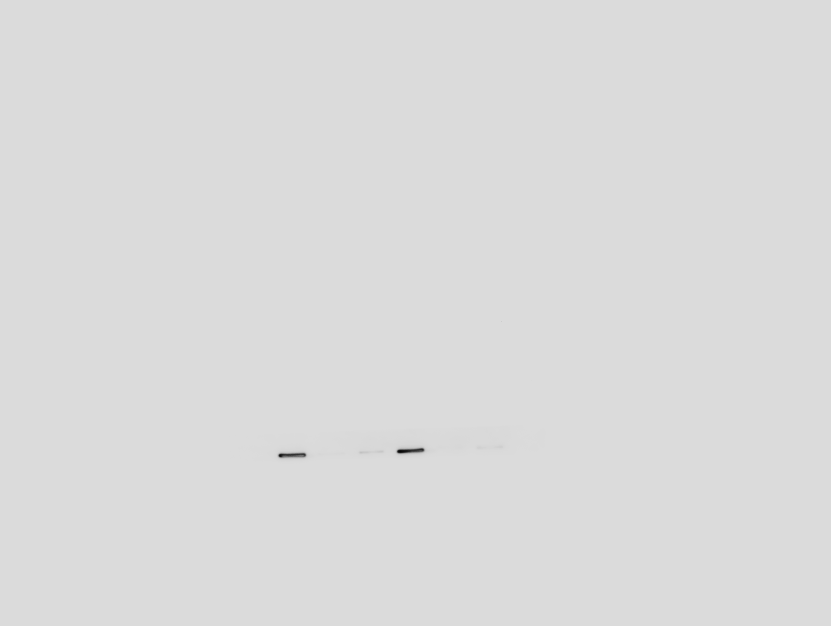


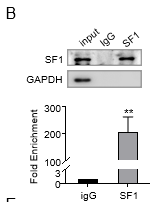


SF1


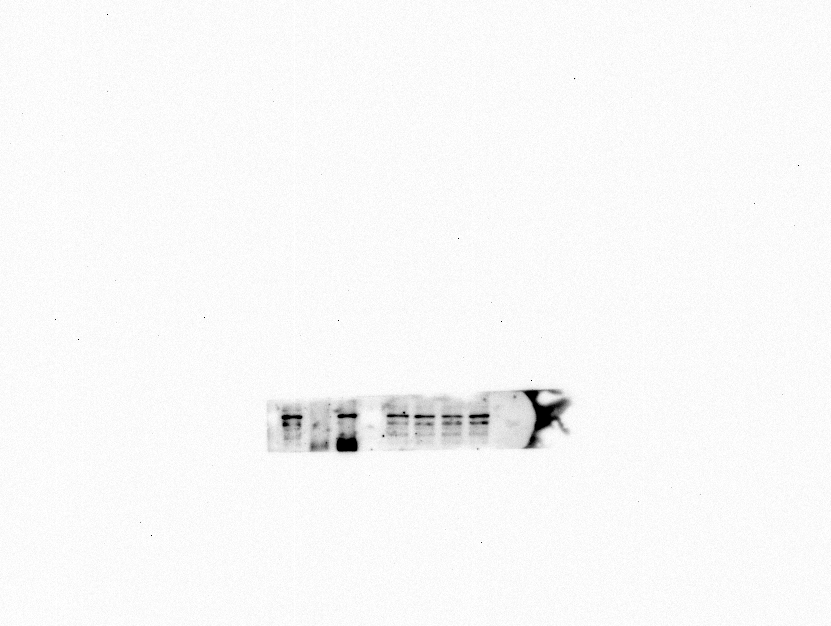


GAPDH


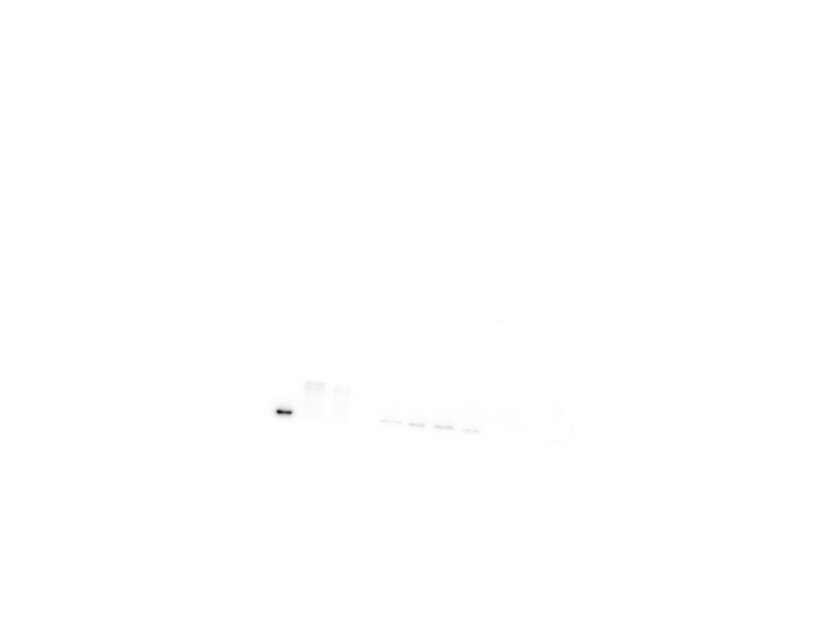


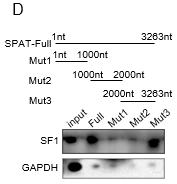


SF1


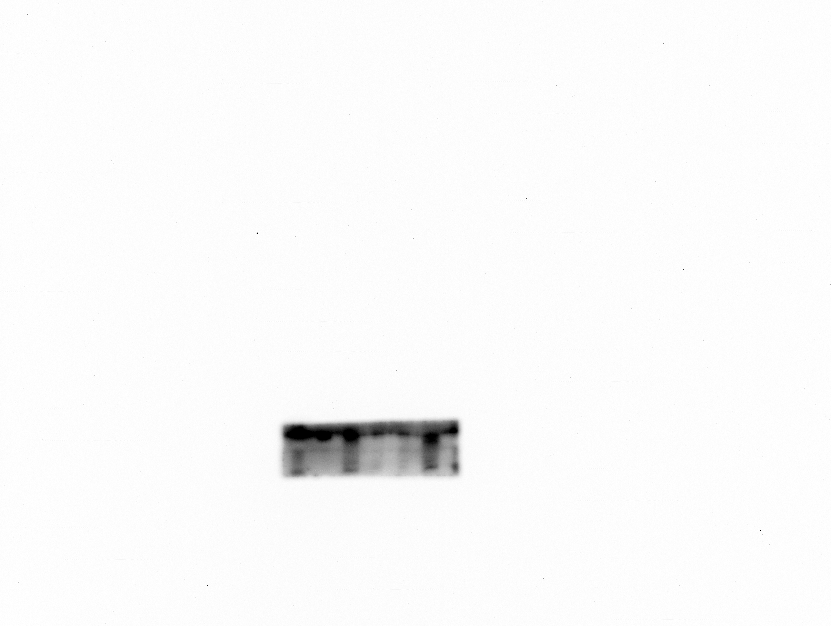


GAPDH


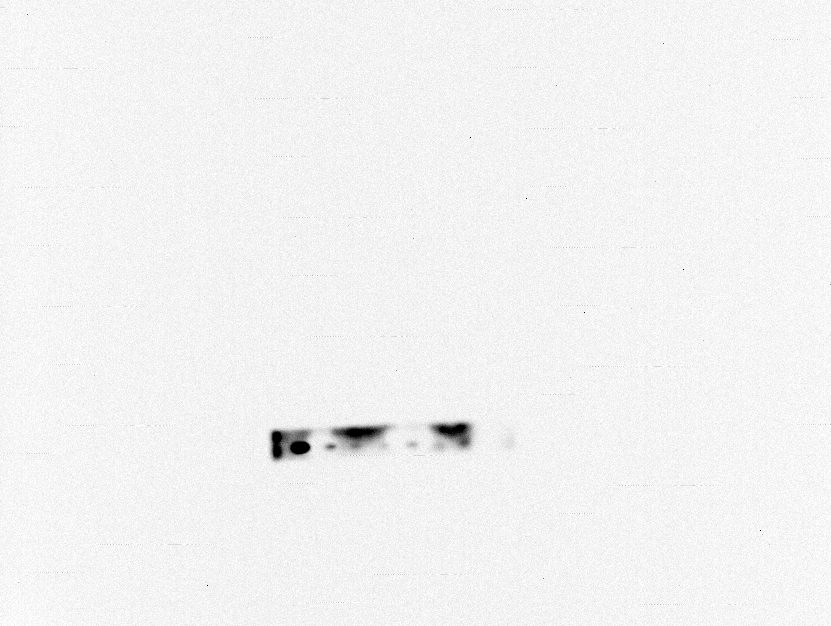


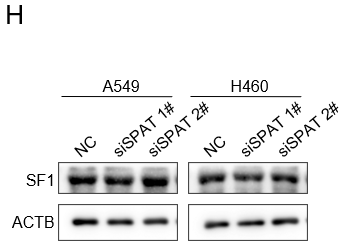


SF1


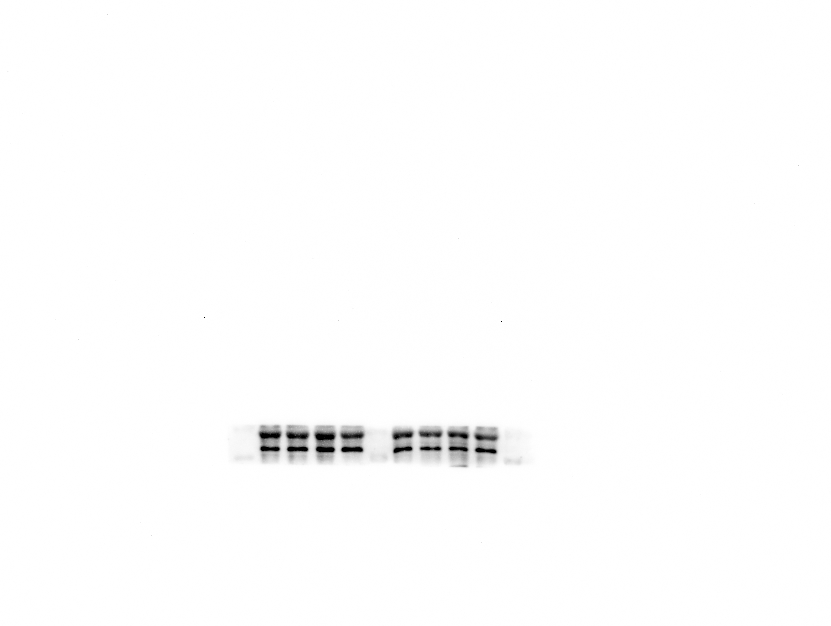


ACTB


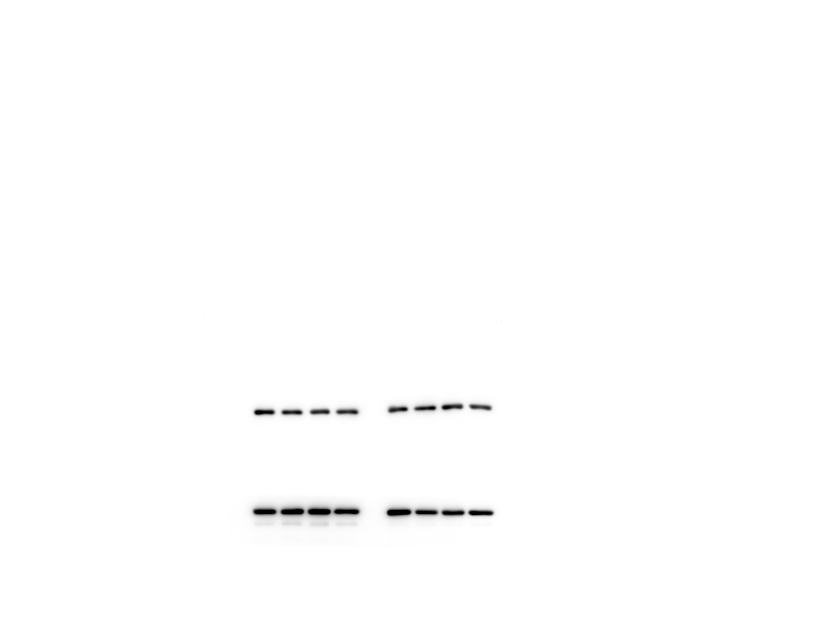


**FIG 5**


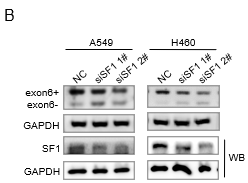


SF1


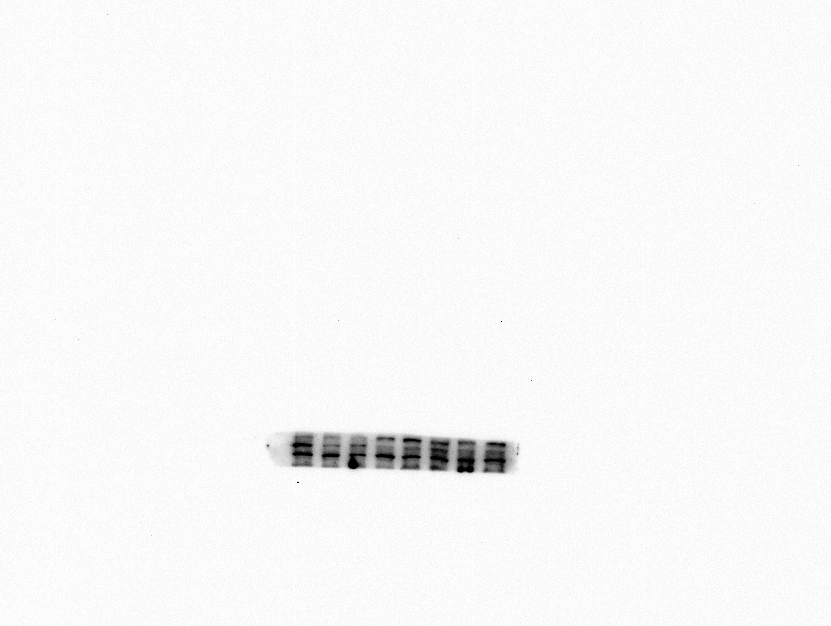


GAPDH


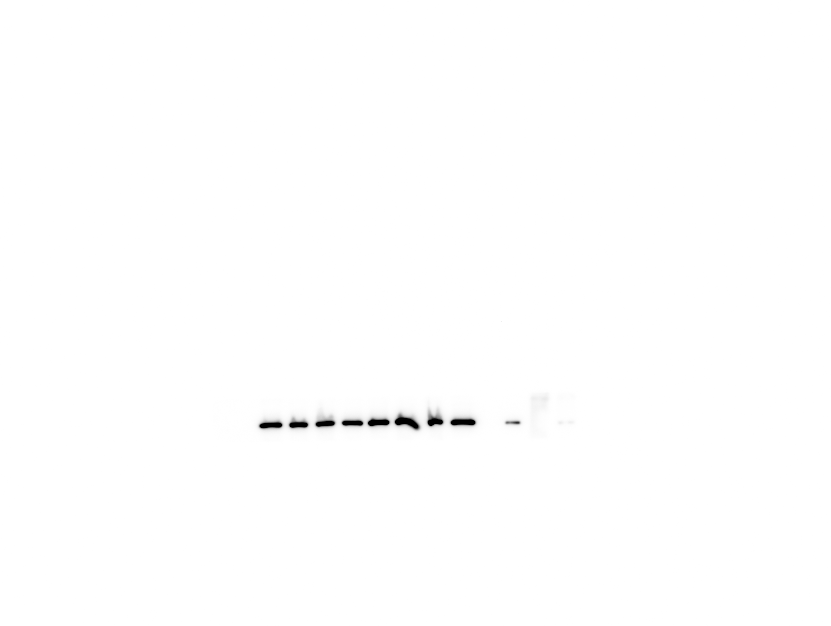


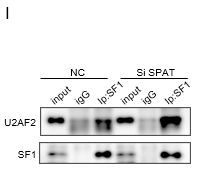


U2AF2


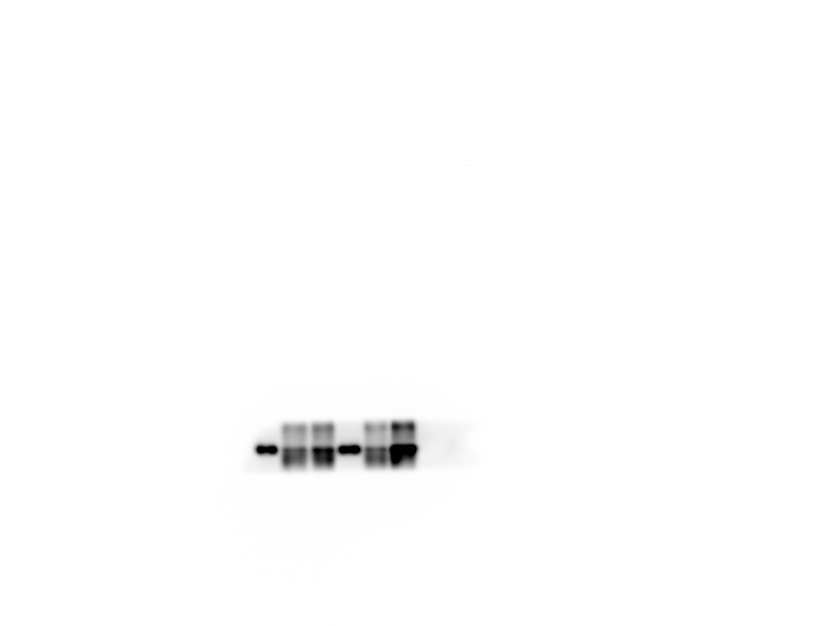


SF1


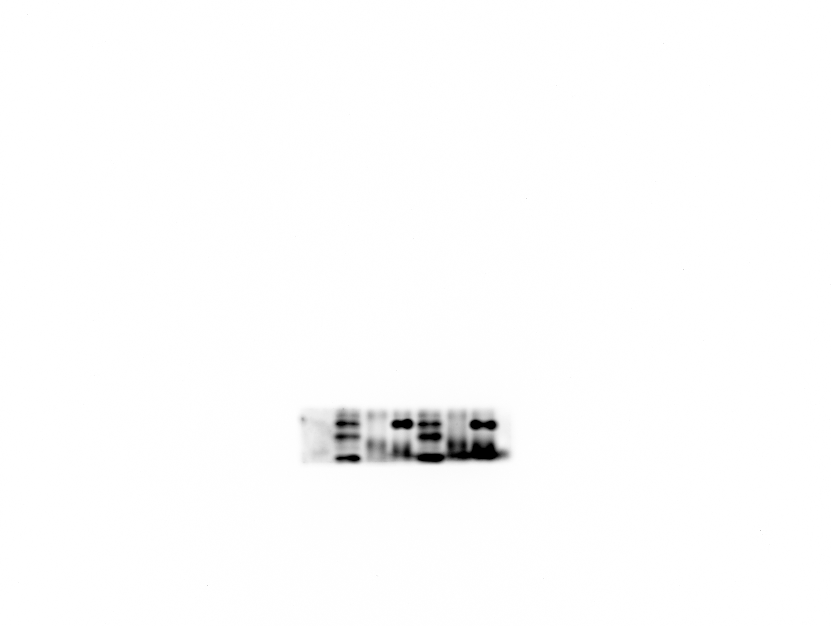


**FIG S6**


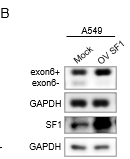


SF1


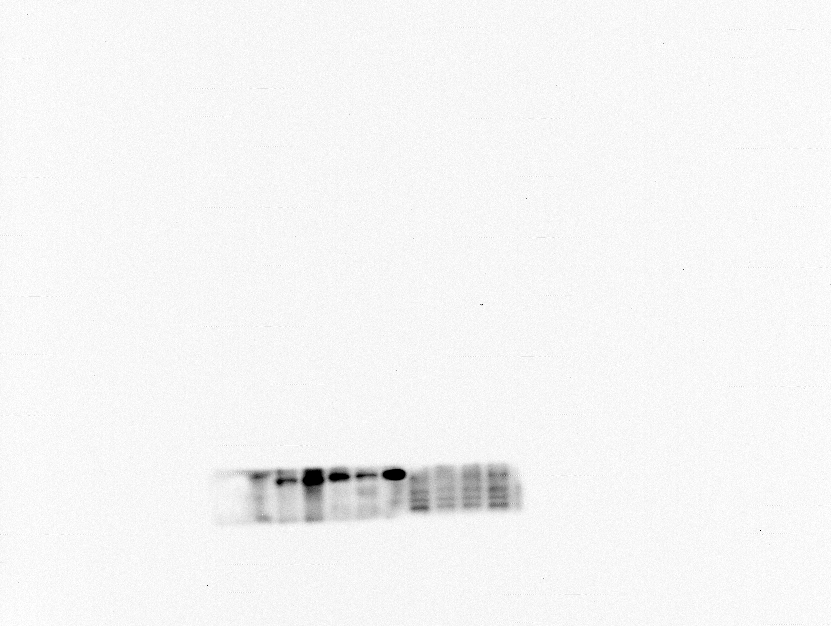


GAPDH


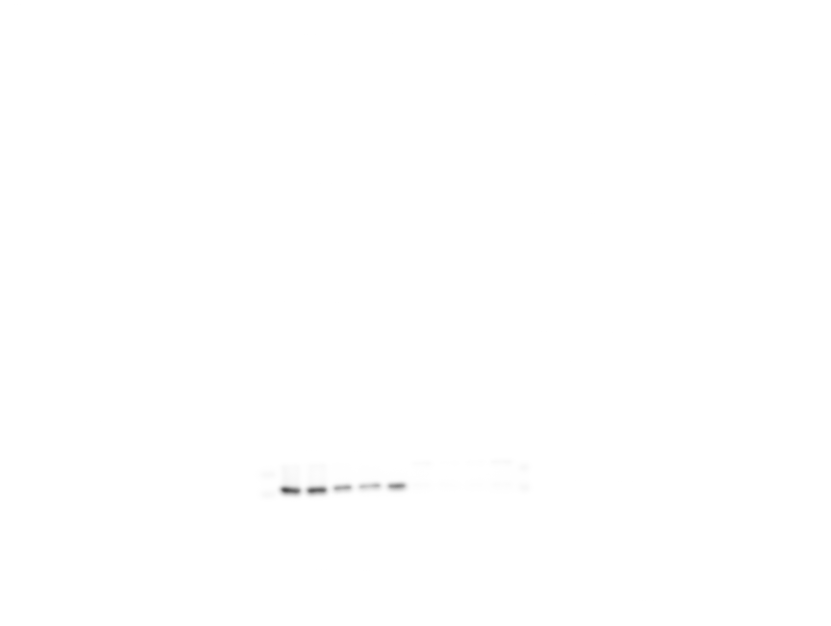


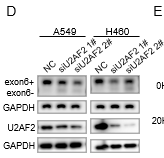


U2AF2


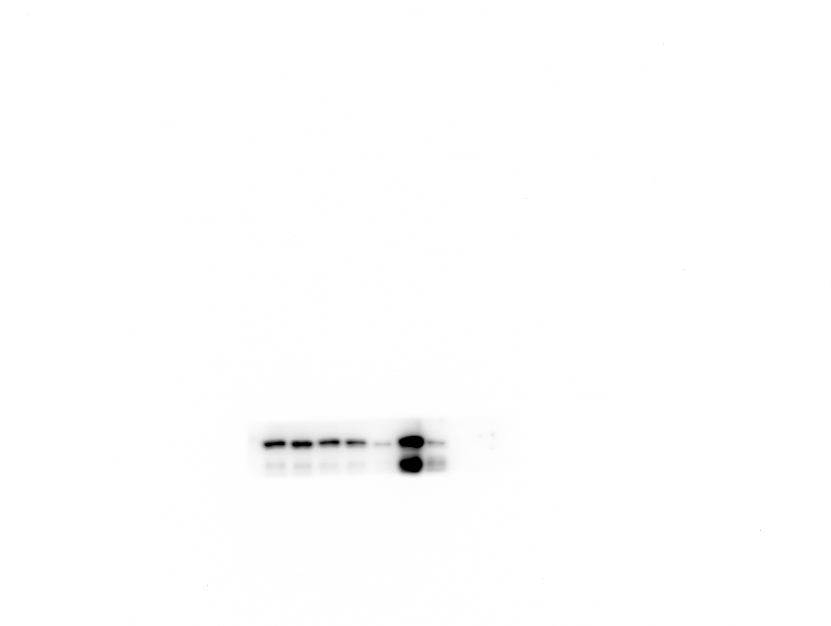


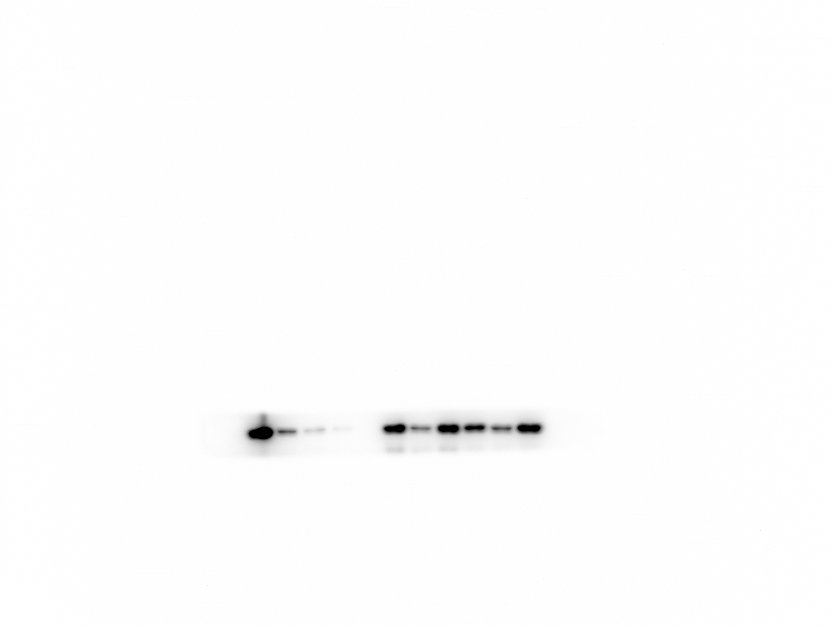


GAPDH


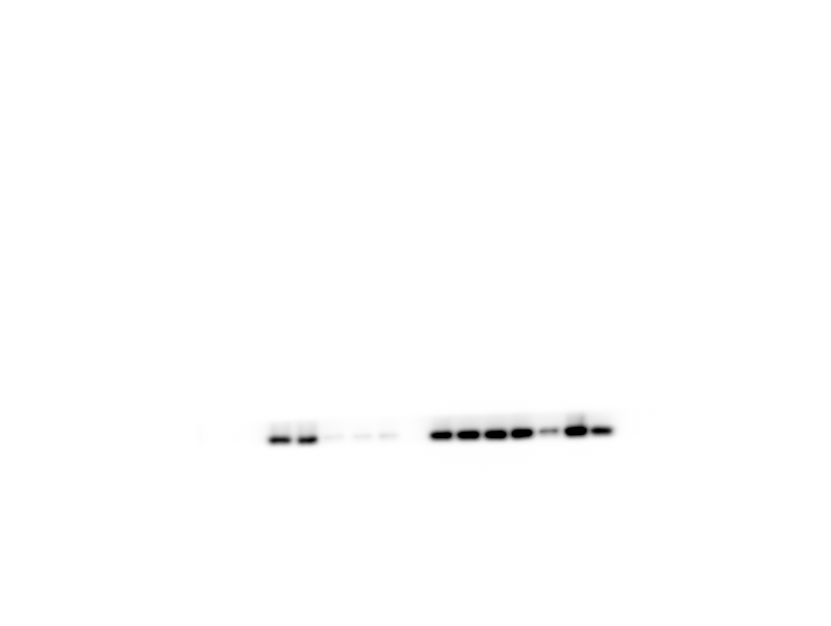


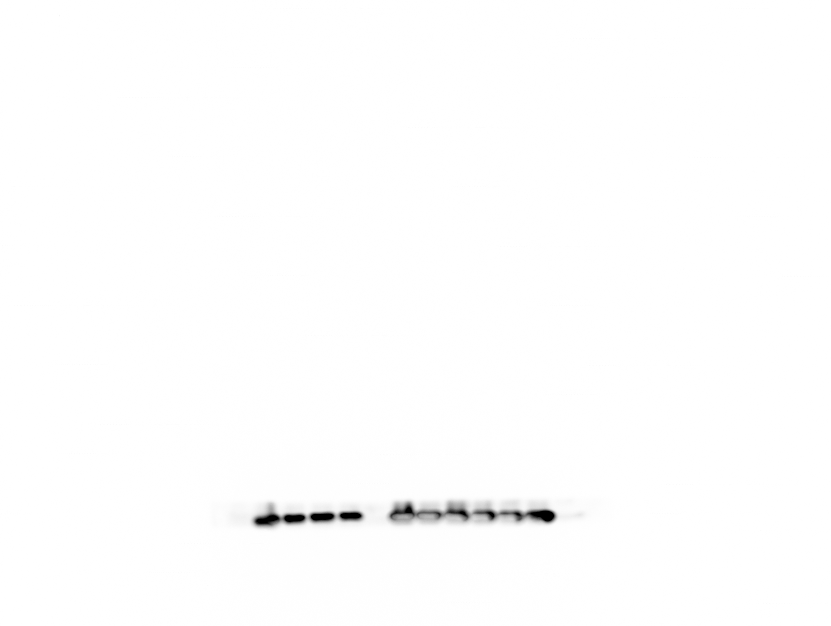


**FIG 7**

**G**


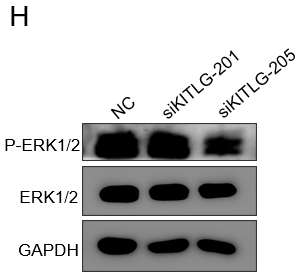


P-ERK1/2


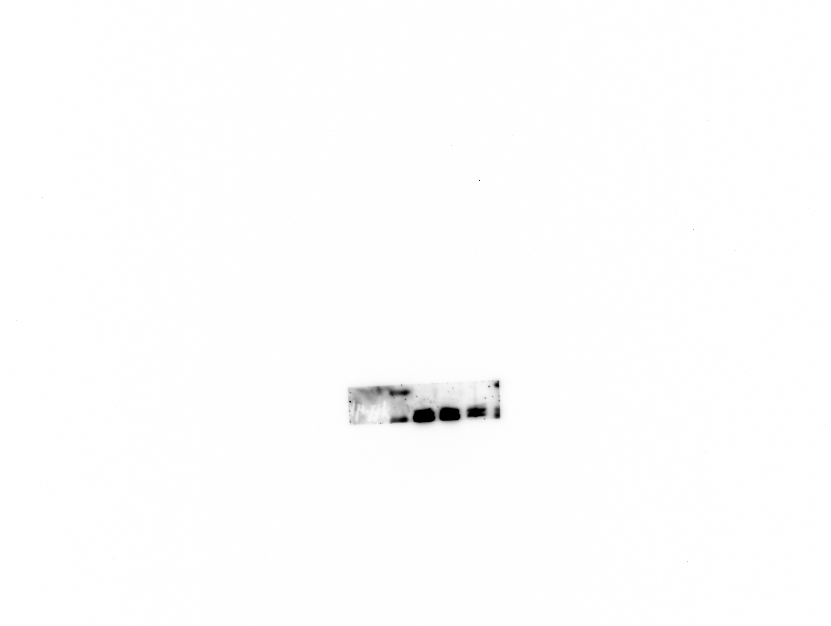


ERK1/2


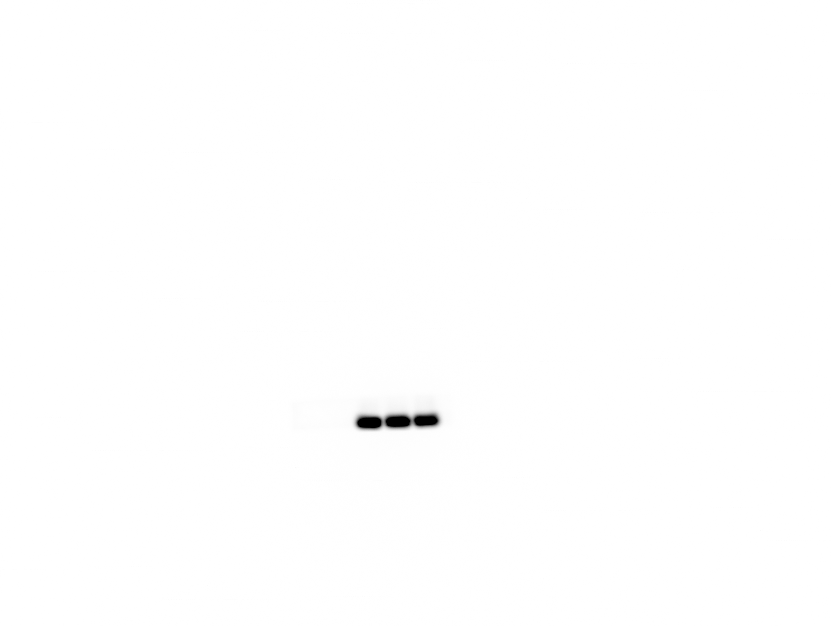


GAPDH


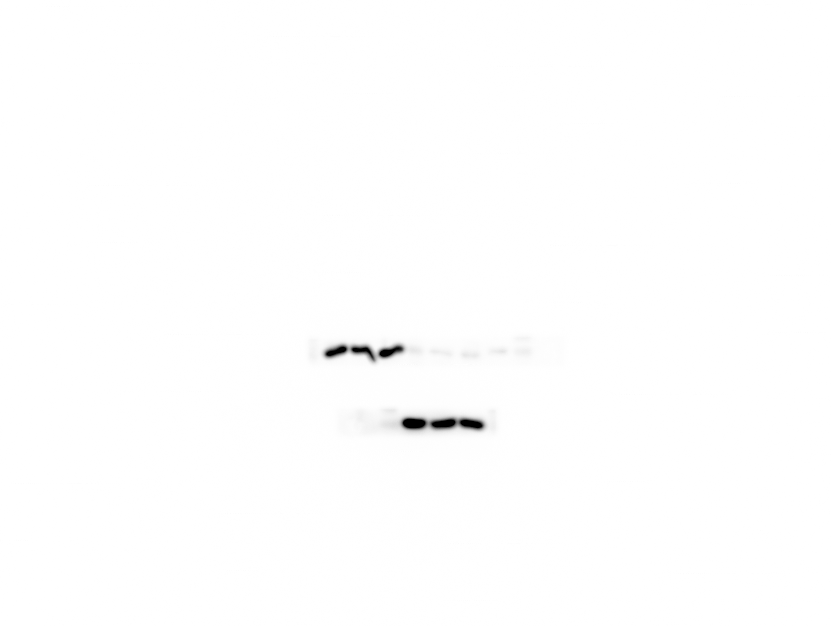


**H**


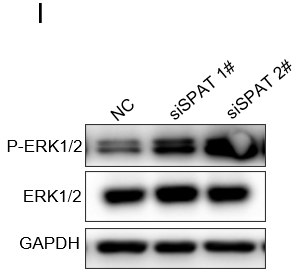


P-ERK1/2


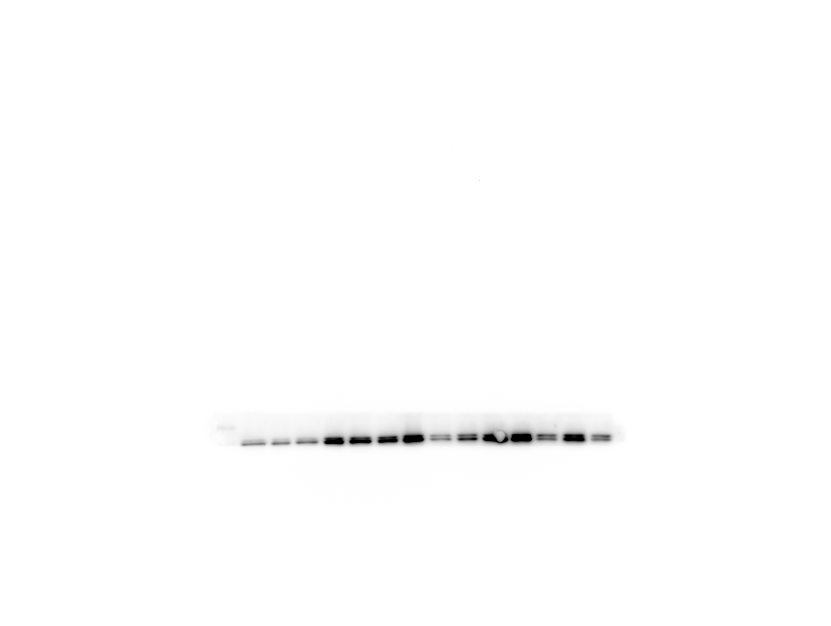


ERK1/2


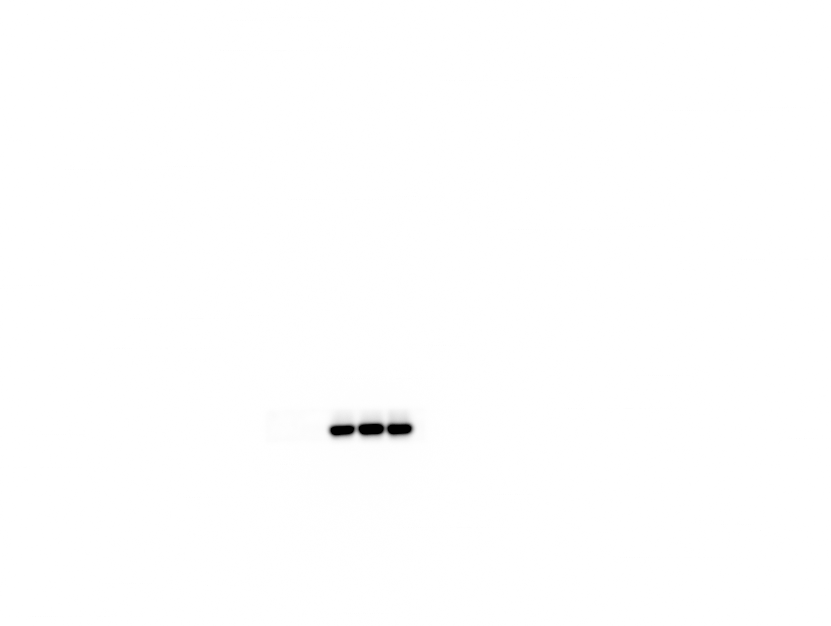


GAPDH


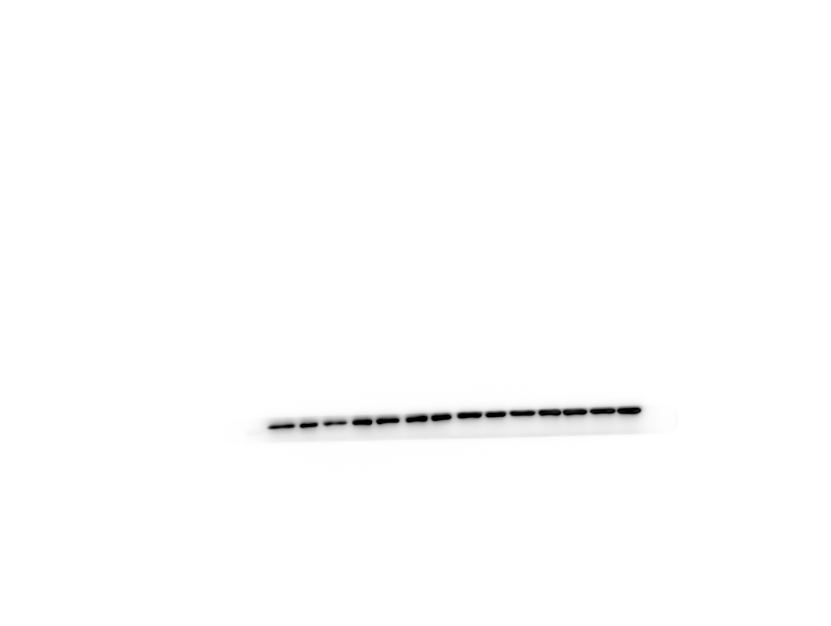


**I**


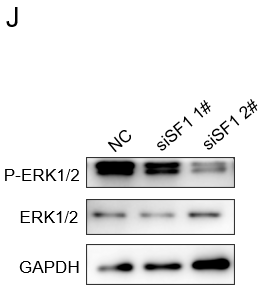


P-ERK1/2


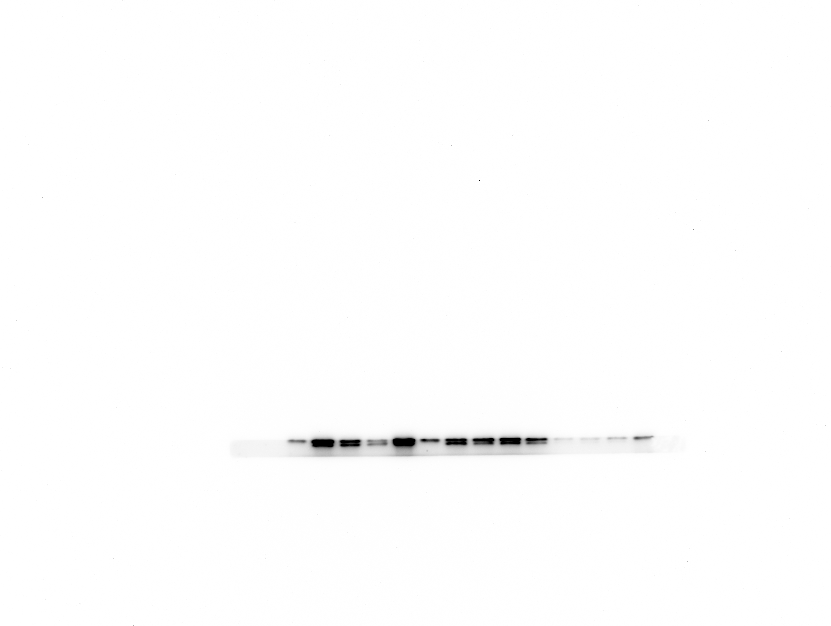


ERK1/2


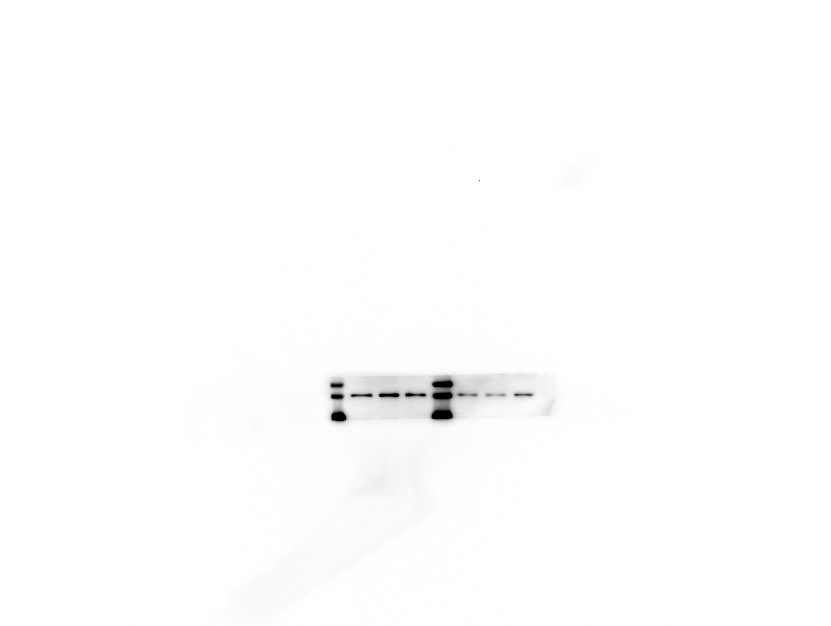


GAPDH


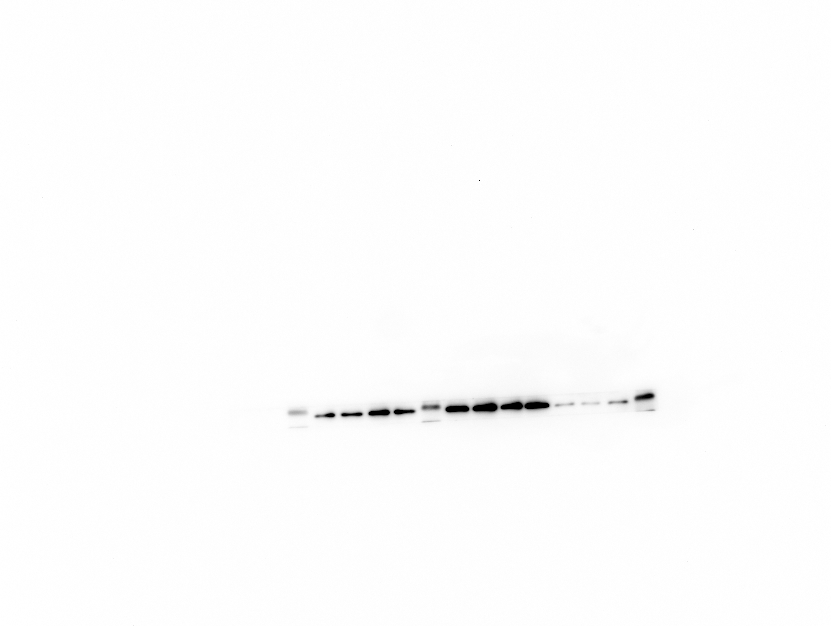


Supplementary Material: Original qPCR data

Fig 1C


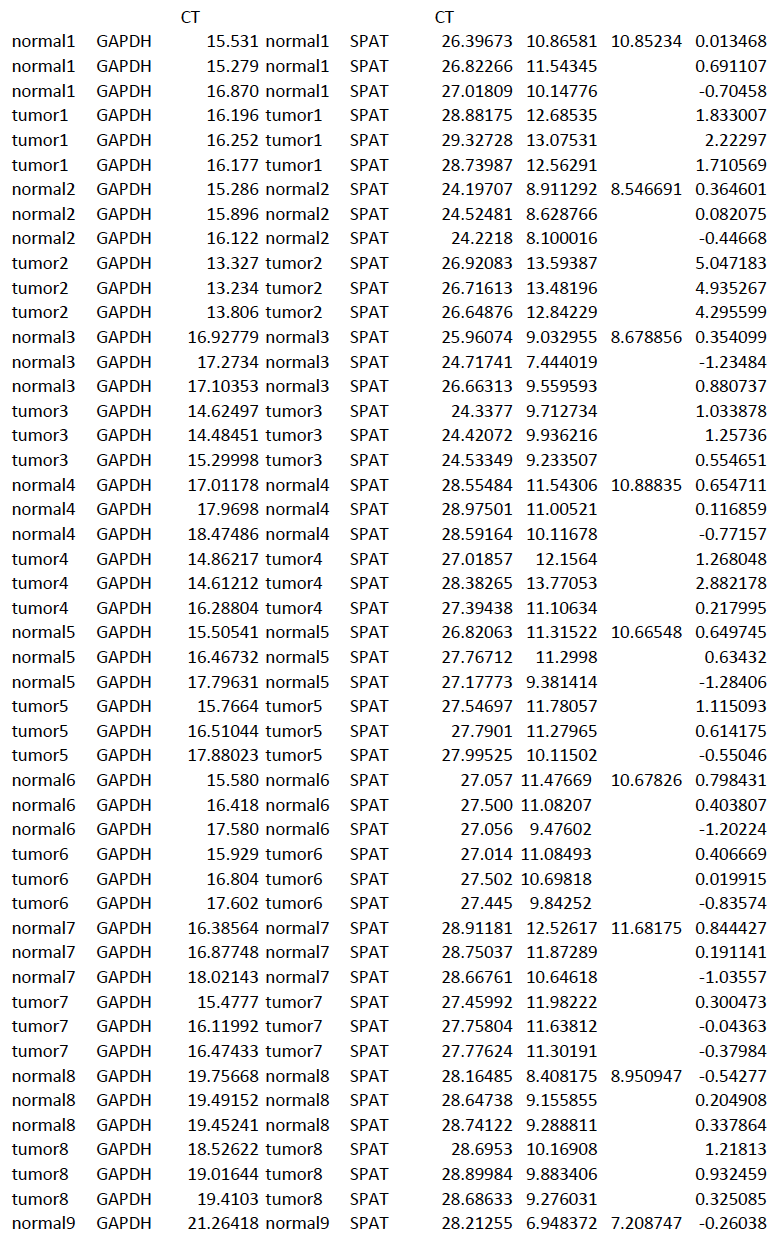


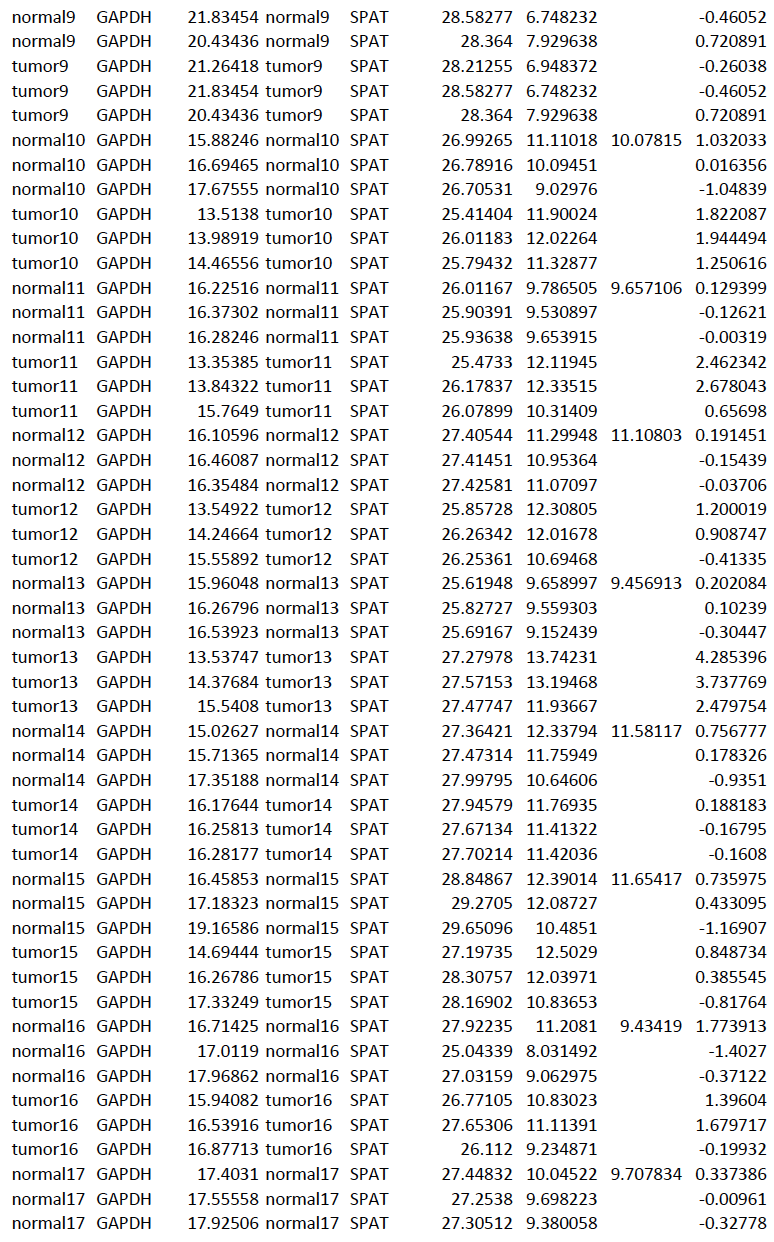


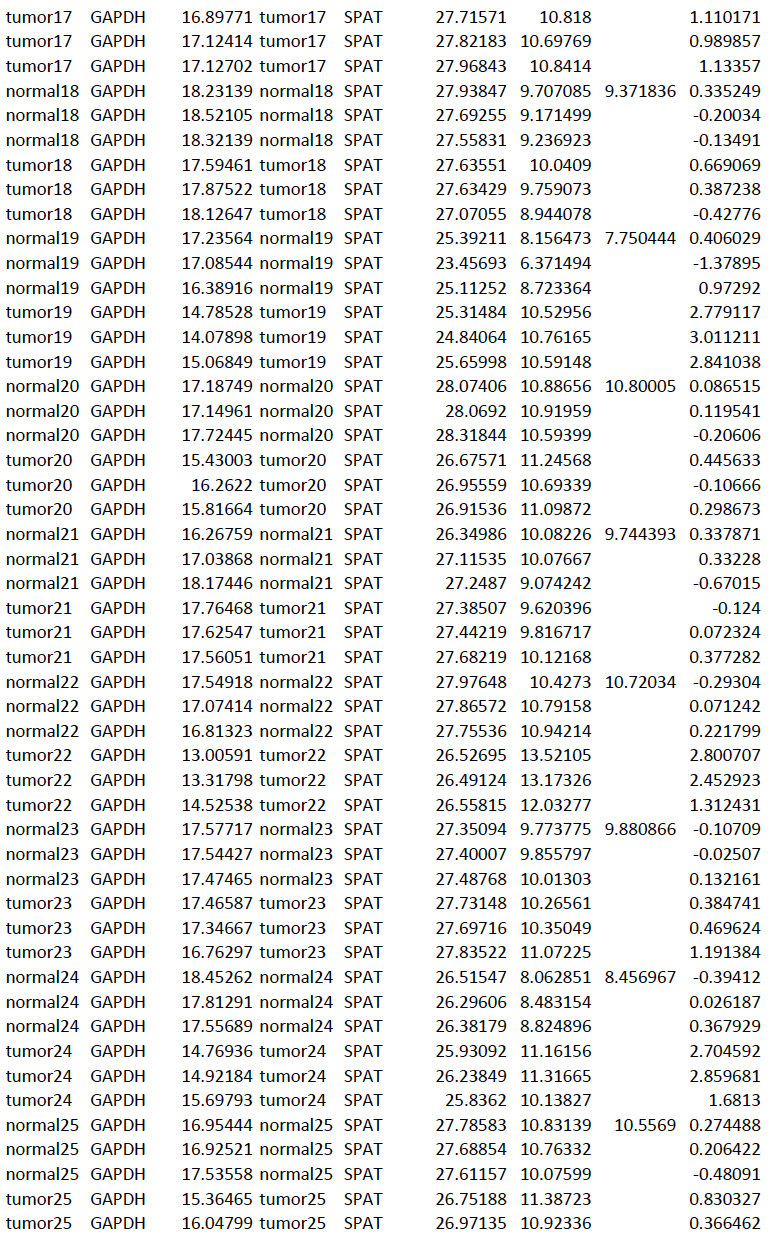

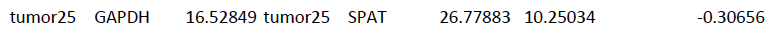


Fig 1F


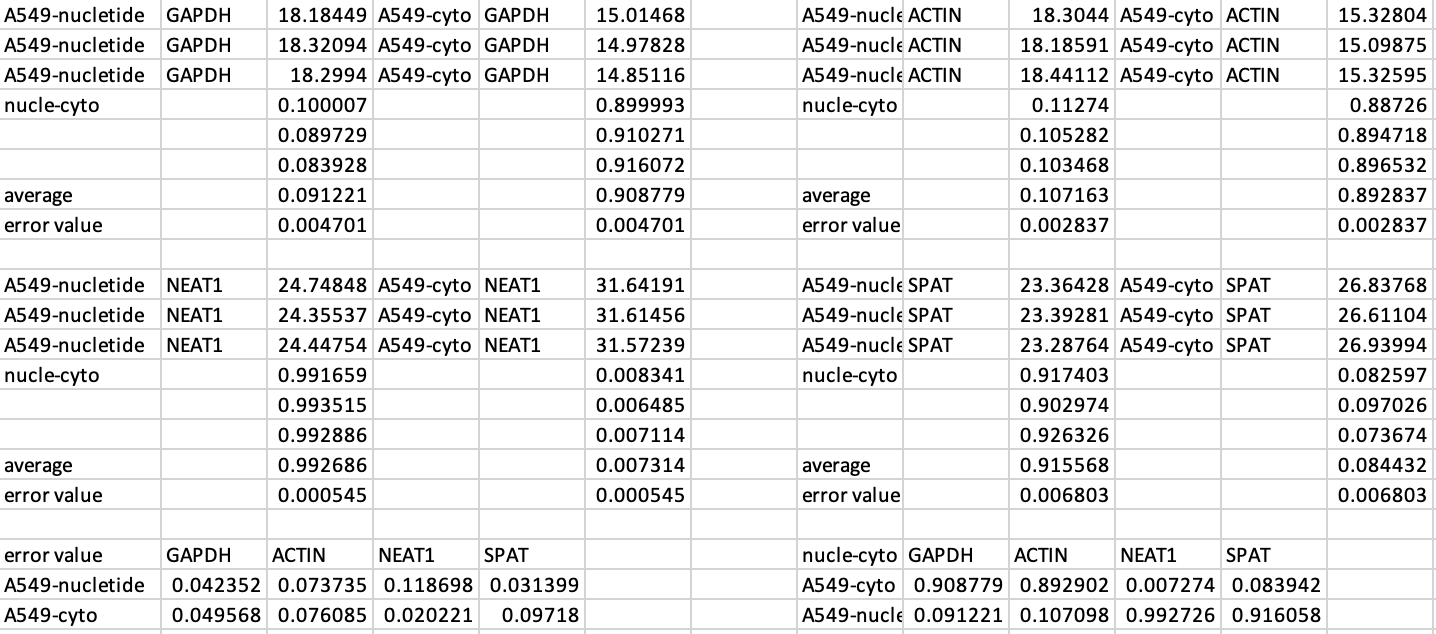


Fig 2A


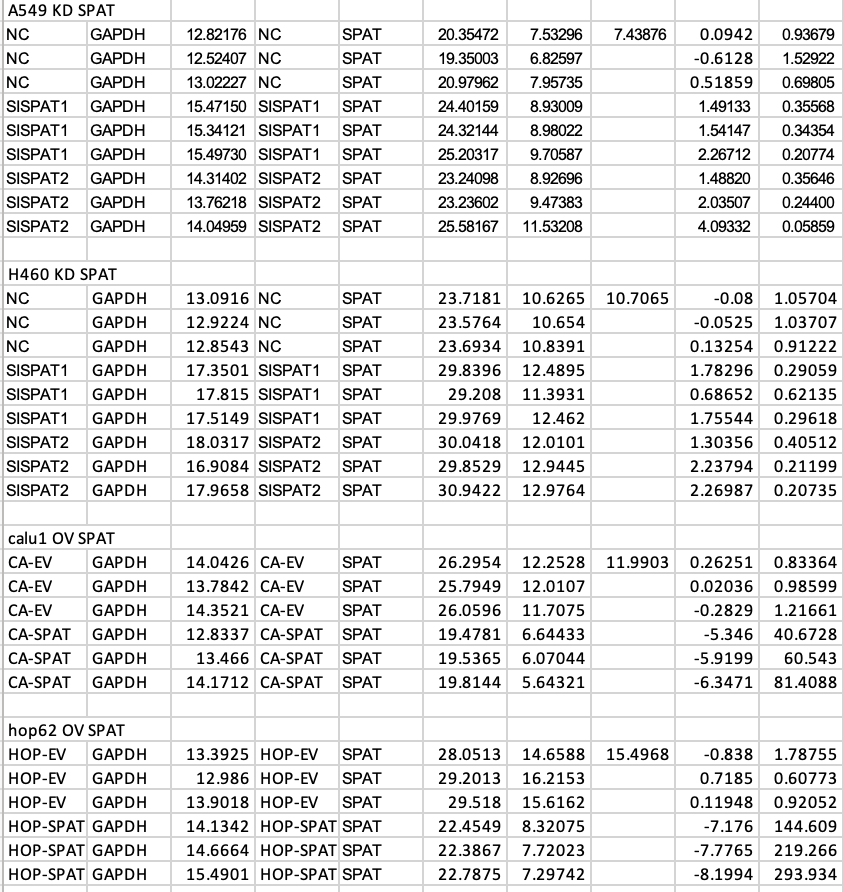


Fig 3B


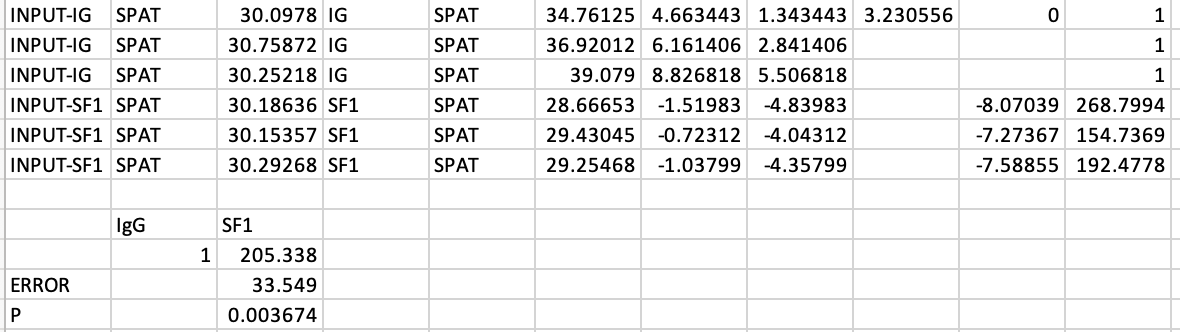


Fig 3F


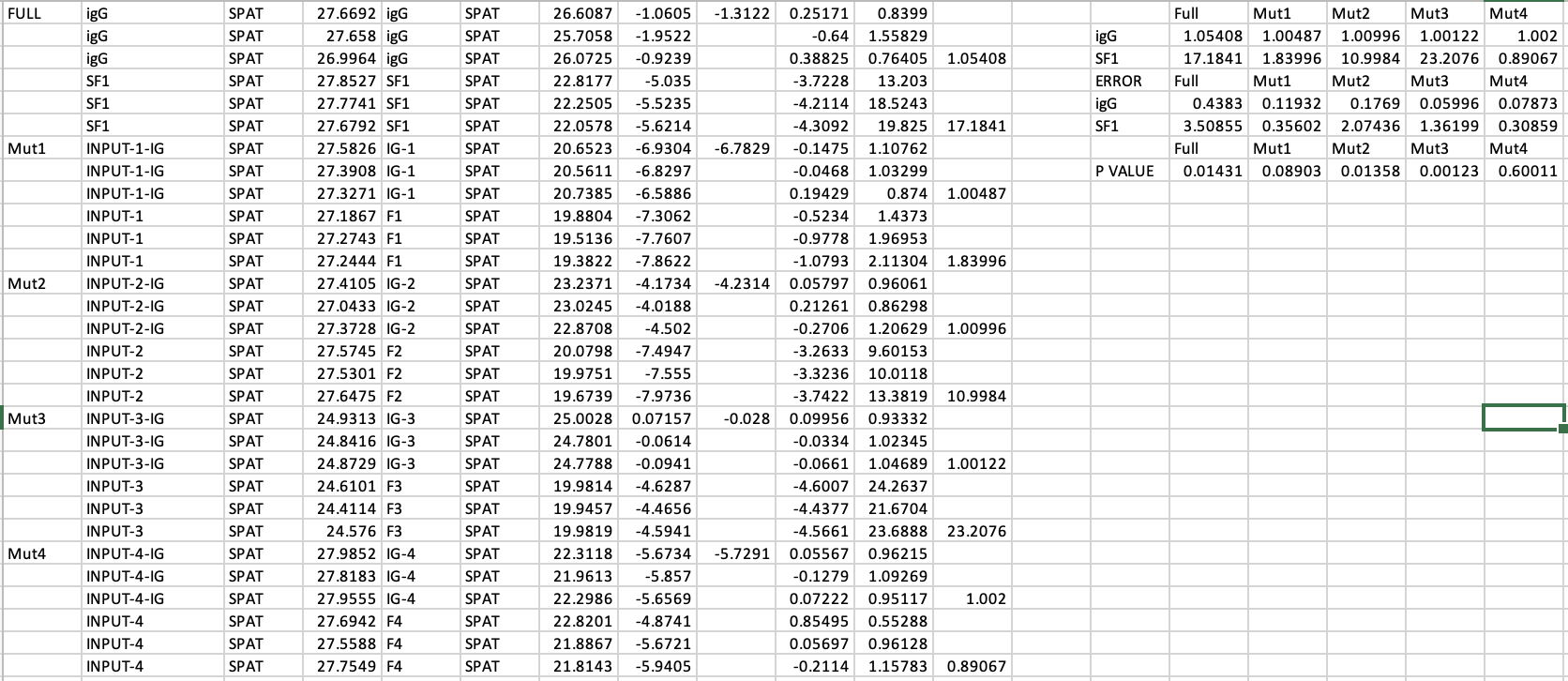


Fig 4D


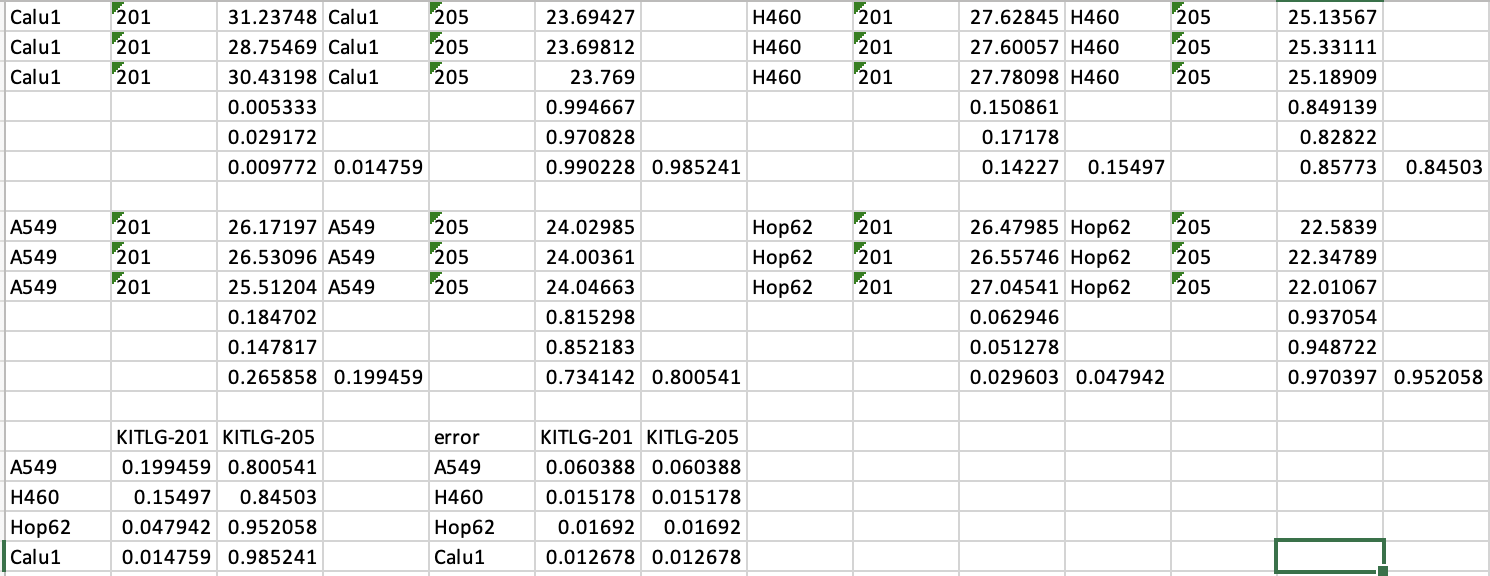


Fig 4F


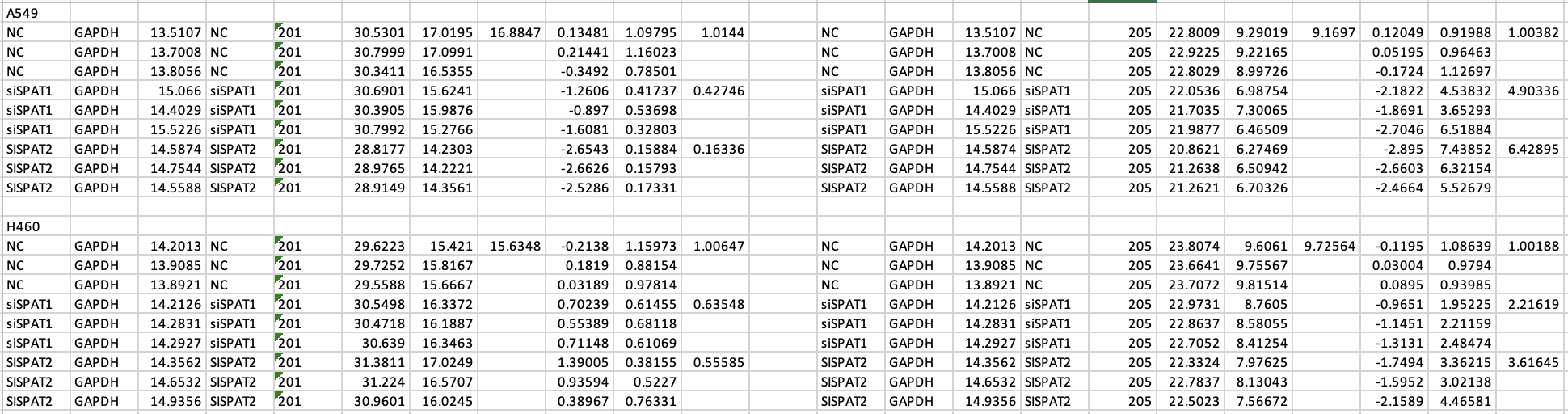


Fig 4H


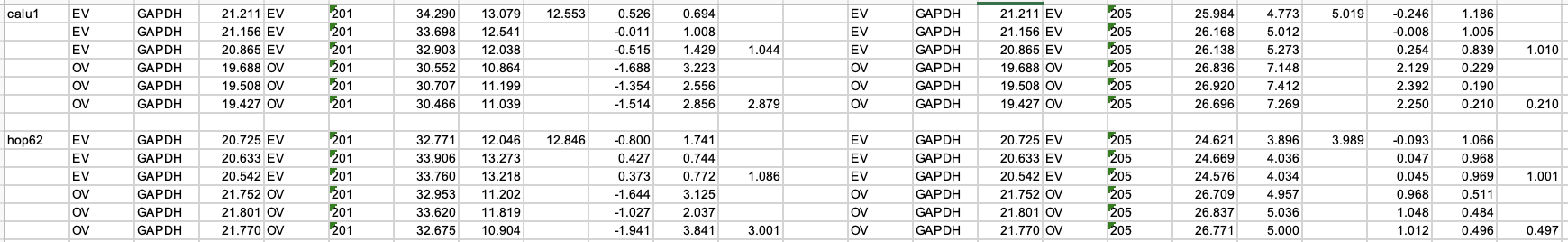


Fig 6D


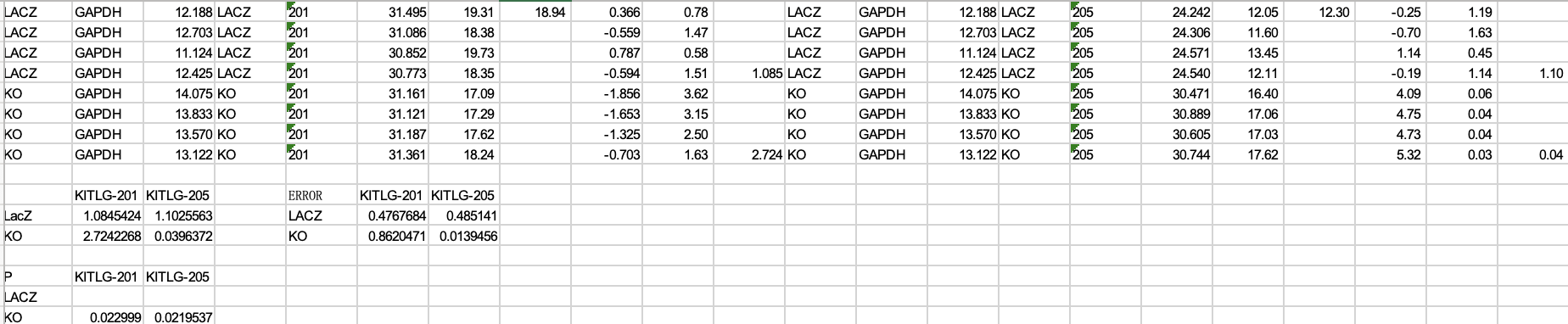


Fig 7D


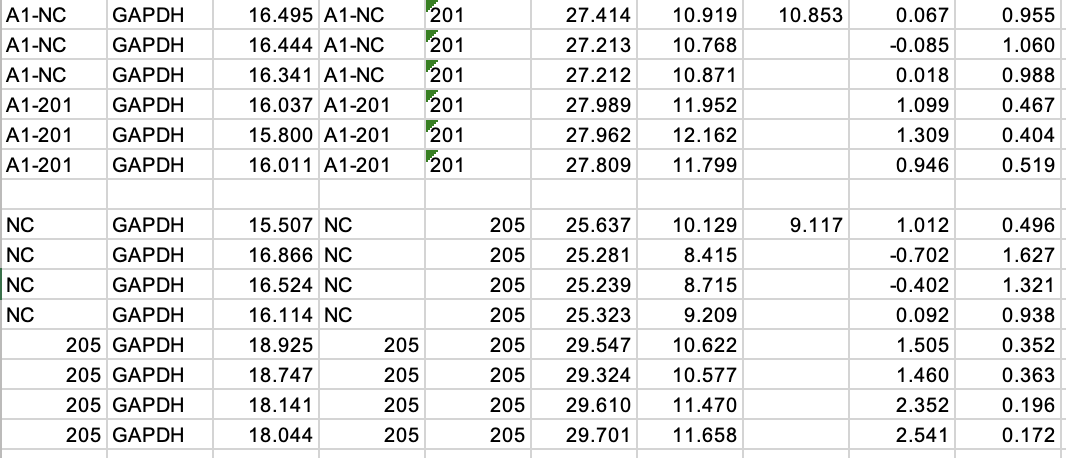


Fig S1C


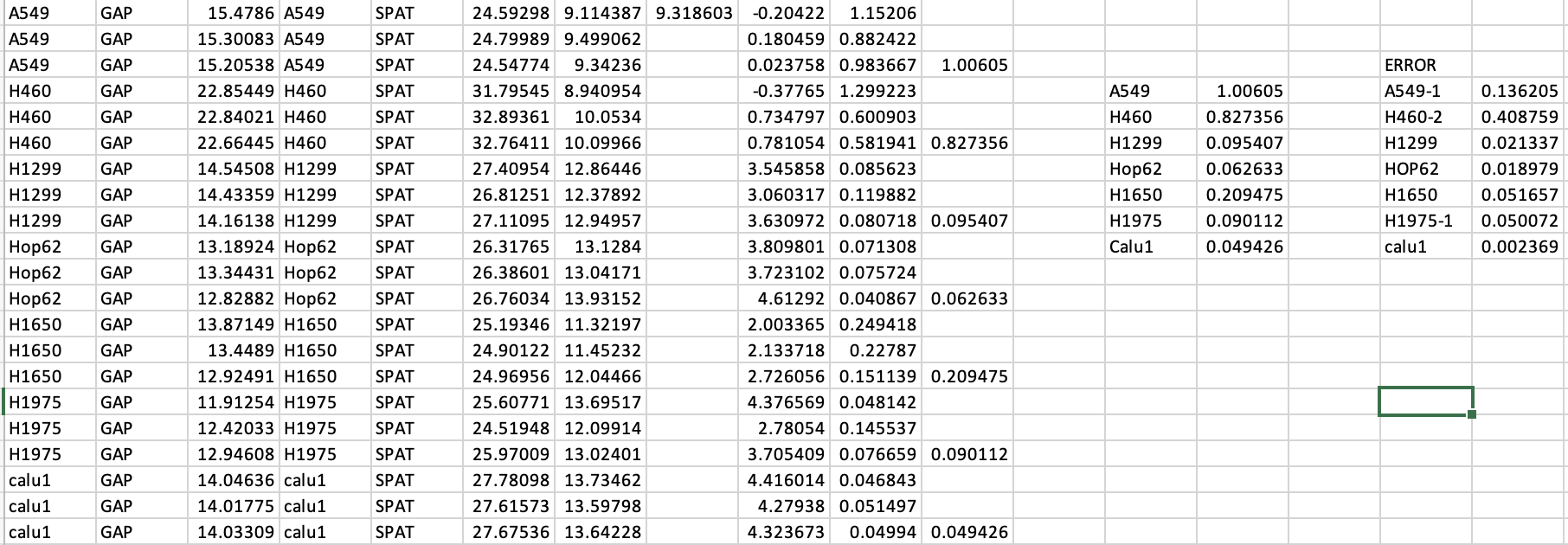


Fig S2A


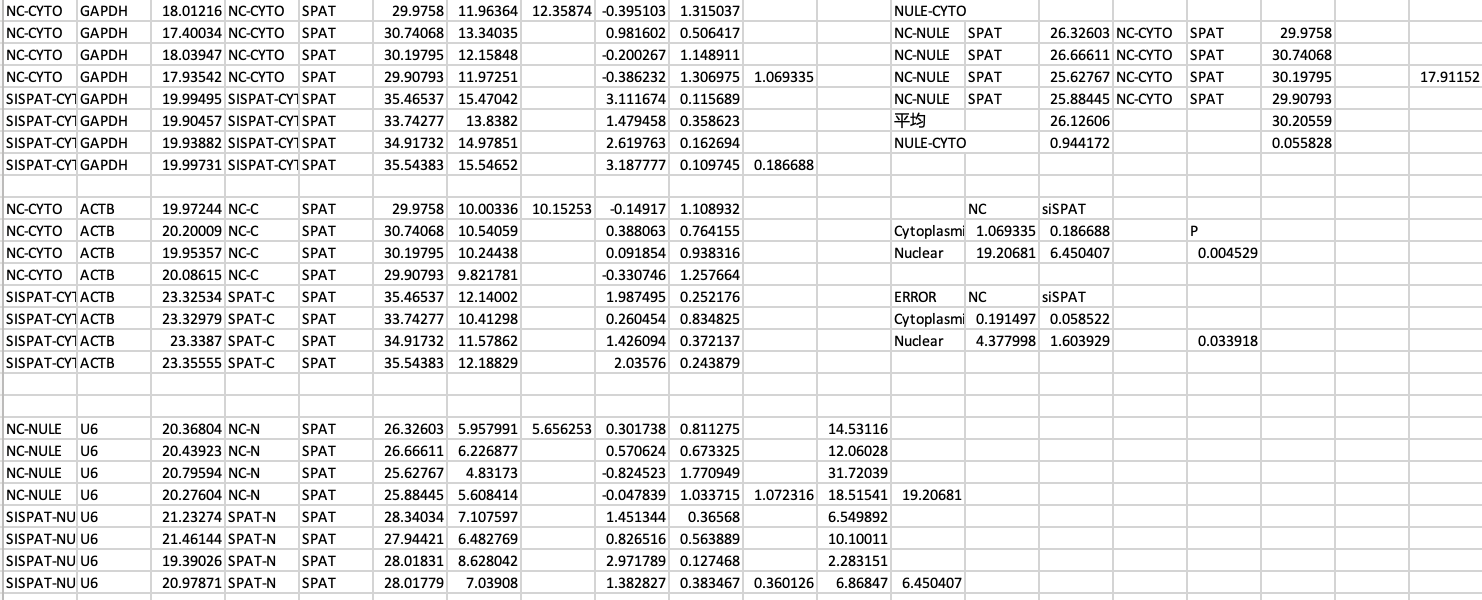


FIG S2D,S2E,S2F


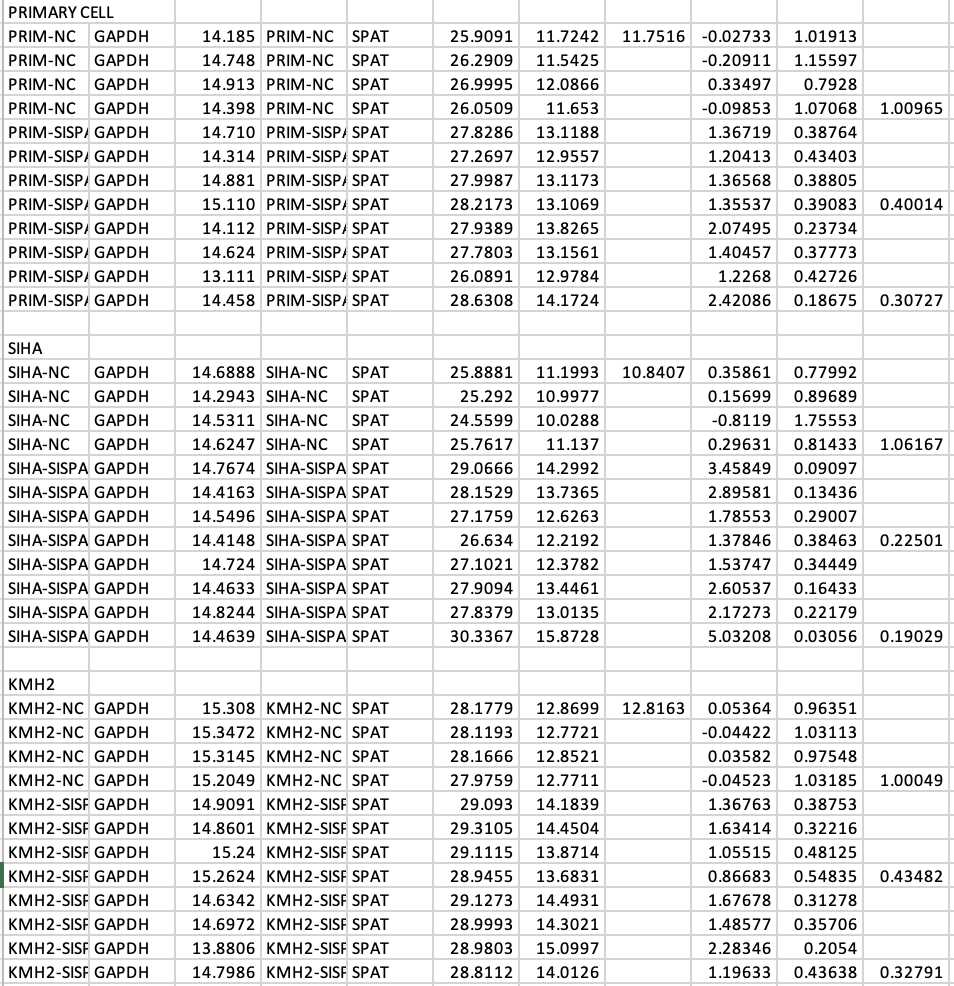


Fig S4C


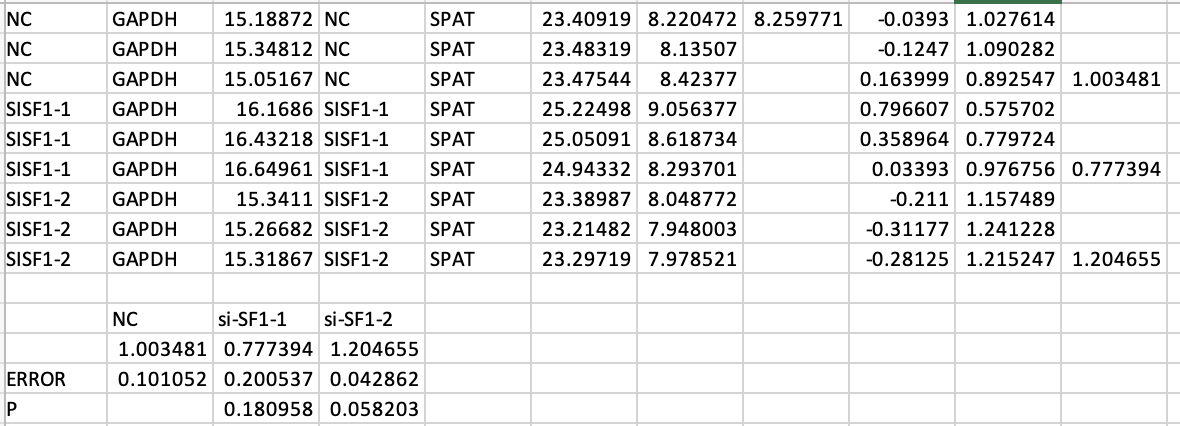


Fig S5B


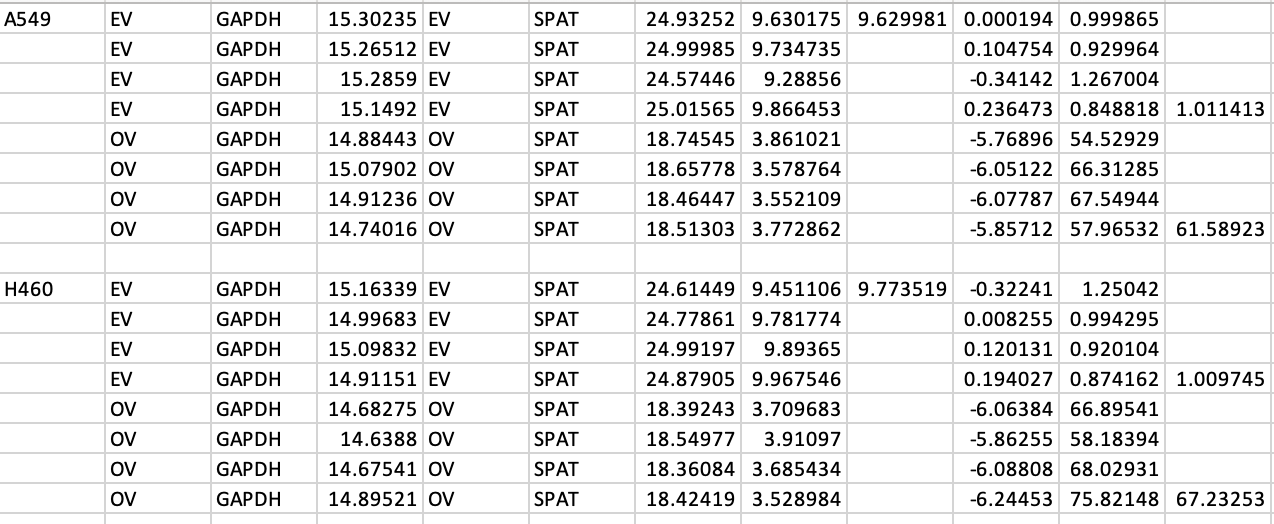


Fig S5D


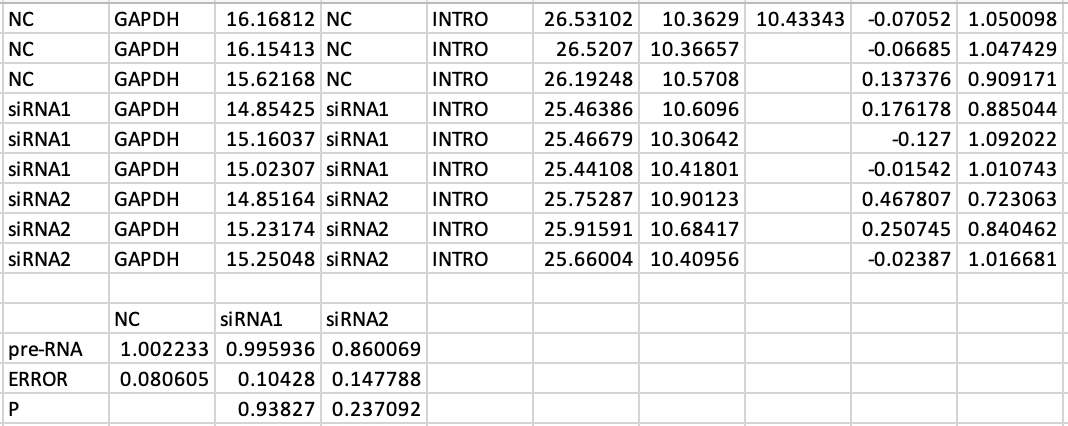


Fig S6A


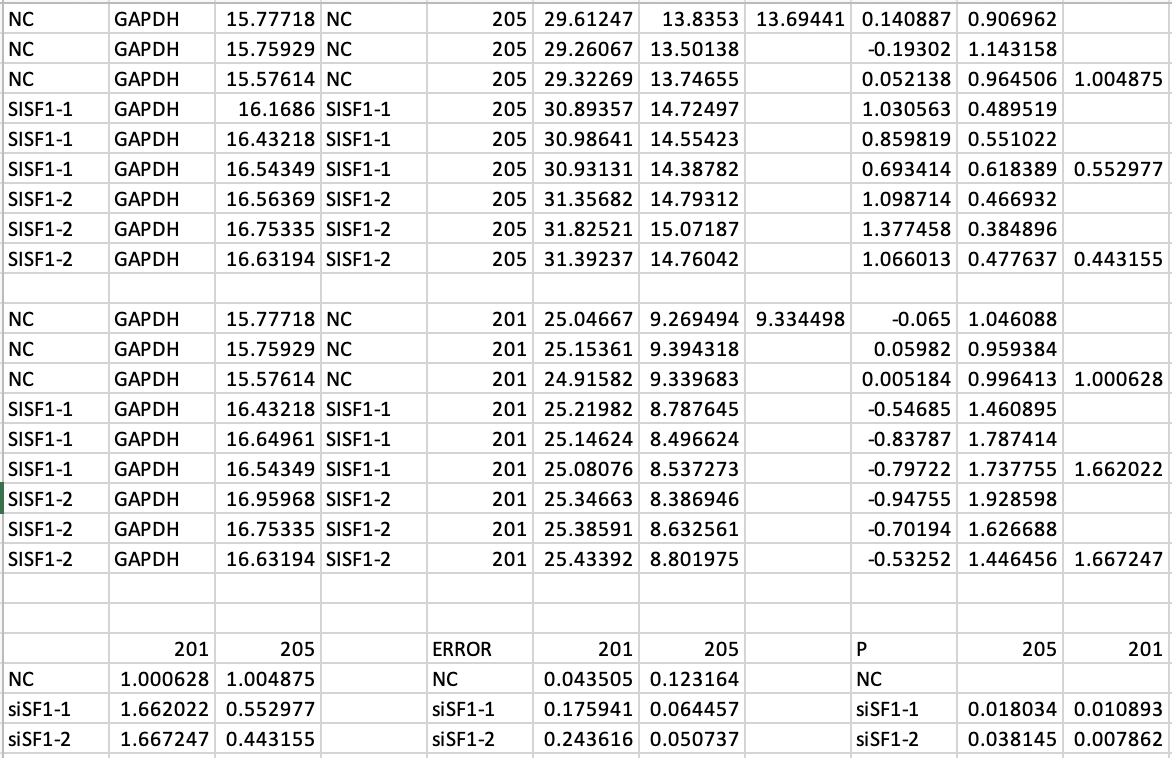

Supplement: Supplementary file 2 — Original data [file 41419_2025_7924_MOESM2_ESM.docx]
